# A New Family of Secreted Toxins in Pathogenic Neisseria Species

**DOI:** 10.1371/journal.ppat.1004592

**Published:** 2015-01-08

**Authors:** Anne Jamet, Agnès B. Jousset, Daniel Euphrasie, Paulette Mukorako, Alix Boucharlat, Alexia Ducousso, Alain Charbit, Xavier Nassif

**Affiliations:** 1 Institut Necker Enfants-Malades, INSERM U1151, Equipe 11, Paris, France; 2 Université Paris Descartes; Sorbonne Paris Cité, Faculté de Médecine, Paris, France; 3 Assistance Publique – Hôpitaux de Paris, Hôpital Necker Enfants Malades, Paris, France; Vanderbilt University, United States of America

## Abstract

The genus *Neisseria* includes both commensal and pathogenic species which are genetically closely related. However, only meningococcus and gonococcus are important human pathogens. Very few toxins are known to be secreted by pathogenic *Neisseria* species. Recently, toxins secreted via type V secretion system and belonging to the widespread family of contact-dependent inhibition (CDI) toxins have been described in numerous species including meningococcus. In this study, we analyzed loci containing the *maf* genes in *N. meningitidis* and *N. gonorrhoeae* and proposed a novel uniform nomenclature for maf genomic islands (MGIs). We demonstrated that *mafB* genes encode secreted polymorphic toxins and that genes immediately downstream of *mafB* encode a specific immunity protein (MafI). We focused on a MafB toxin found in meningococcal strain NEM8013 and characterized its EndoU ribonuclease activity. *maf* genes represent 2% of the genome of pathogenic *Neisseria*, and are virtually absent from non-pathogenic species, thus arguing for an important biological role. Indeed, we showed that overexpression of one of the four MafB toxins of strain NEM8013 provides an advantage in competition assays, suggesting a role of *maf* loci in niche adaptation.

## Author Summary

Many bacteria are able to secrete toxins targeted against neighboring cells. In order to protect themselves against their own toxin, they also express an “immunity” protein. *In silico* analysis of bacterial genomes predicts that numerous genes could encode potential new toxin-immunity systems. The recently described CDI system is involved in contact-dependent inhibition of growth and confers to its host strain a significant advantage in competitive ecosystems such as the gastro-intestinal tract. Indeed, an *Escherichia coli* CDI+ strain is able to outcompete CDI- strains and to become predominant. Here, we show that a large family of genes called “*maf*”, found in pathogenic *Neisseria* species, encodes a toxin-immunity system. We demonstrate that a toxin named MafB_MGI-1NEM8013_ inhibits the growth of *E. coli* by degrading RNA and show that the immunity protein MafI_MGI-1NEM8013_ is able to abolish the toxicity. MafB toxins exhibit highly variable toxic domains. This variability of secreted toxins could be important to compete against bacteria of different species sharing the same reservoir. Since a strain may contain numerous toxin-immunity systems that can all play a role in interbacterial competition, deciphering interactions between these systems will allow a better understanding of complex bacterial communities.

## Introduction

The growing number of sequenced bacterial genomes has led to the computer-based prediction of numerous novel bacterial factors possibly involved in virulence. As a result, many novel putative bacterial toxins have been identified by sequence-homology criteria. However, very few of these bacterial proteins have been tested for their toxic activity. Using *in silico* analysis, Aravind and colleagues have recently described widespread genes encoding putative secreted multi-domain toxins grouped under the name of bacterial polymorphic toxin systems (or polymorphic toxin-immunity systems) [1]–[3]. *In silico* analysis identified over 150 distinct toxin domains in these systems including many putative peptidase, nuclease or deaminase domains. Immunity genes found immediately downstream of the toxin genes encode highly variable proteins that protect bacteria from their own toxins or from toxins secreted by neighboring cells [4]–[6]. Immunity genes are a characteristic of polymorphic toxin systems that distinguishes them from host-directed toxins (*i.e.* cholera toxin or pertussis toxin) [7]. The polymorphic toxin systems are typically encoded on hypervariable chromosomal islands with characteristics of horizontal gene transfer [1]. These systems are found in both Gram- negative and positive bacteria [8]. The dominant hypothesis is that polymorphic toxin systems are primarily involved in conflict between related bacterial strains. The N-terminal domain of the toxin is typically related to trafficking mode whereas the C-terminal domain carries the toxic activity [1]. In a defined family of polymorphic toxins, the N-terminal domains are similar, while the C-terminal domains are highly variable. Toxins are potentially secreted by Type II, V, VI, or VII (ESX) secretion systems [1]. Toxins are encoded in loci that also contain standalone cassettes and immunity genes. Cassettes encoding alternative C-termini could promote diversity of toxic activities through genetic recombination [1], [9], [10].

The recently described contact-dependent growth inhibition (CDI) systems are a subgroup of polymorphic toxin systems [1], [11]. Toxins encoded by CDI systems are large filamentous proteins that exhibit RHS (rearrangement hotspot) or filamentous haemagglutinin repeats in their central region [4], [6], [8]. Rhs proteins are likely to be exported through the Type VI secretion machinery [6], whereas toxins with filamentous haemagglutinin repeats are exported through the Type 5 Secretion System (T5SS) [8], [12]. The first CDI toxin secreted by a T5SS was reported in *Escherichia coli* EC93 (CdiA^EC93^) [11]. *E. coli* EC93 was found to inhibit the growth of other *E. coli* strains (*i.e. E. coli* K-12) in co-culture experiments. The growth inhibition mediated by *E. coli* EC93 required a direct contact between toxic and target cells. In CDI systems, the toxin CdiA is secreted by an outer membrane transporter named CdiB. CdiA and CdiB are part of a two-partner secretion protein family (type Vb). Subsequently, several studies have demonstrated that CDI systems are present in many species including *Neisseria meningitidis* [4], [5], [13]. Moreover, it has been recently demonstrated that the two-partner system TpsAB is indeed a functional CDI system in *N. meningitidis* strain B16B6 [14].

In addition to non-pathogenic commensal species, the genus *Neisseria* includes two human pathogens: *N. gonorrhoeae* (the gonococcus) and *N. meningitidis* (the meningococcus). *N. gonorrhoeae* colonizes the uro-genital tract and is a common cause of sexually transmitted infections [15]. *N. meningitidis* is commonly found in the nasopharynx of healthy individuals, where it can cross the mucosal epithelium and cause sepsis or meningitis [16]. For yet unknown reasons, some meningococcal strains belonging to a limited number of clonal complexes, known as hyper invasive clonal complexes, are much more likely to cause disease than others [17].

Comparison of the genomes of related bacteria that exhibit distinct pathogenic phenotypes can identify relevant genetic variations linked to virulence. The availability of complete genome sequences for several strains of both pathogenic and non-pathogenic species of *Neisseria* genus enabled their *in silico* comparison [18]–[23]. Genes involved in adherence to epithelial cells, in capsule biosynthesis, or in iron uptake are well known to be crucial for pathogenicity [16], [21], [24]. Nevertheless, their presence is not sufficient to explain the invasiveness of pathogenic strains compared to non-pathogenic strains. Thus, to date, genomic comparisons between pathogenic and non-pathogenic *Neisseria* species have failed to identify genes sufficient and necessary to cause disease [18], [21], [25]. The accessory genome, which is composed of genes found only in some strains, confers strain-specific traits and is commonly acquired through horizontal transfer [18]. The accessory genome may be linked to virulence as illustrated by pathogenicity islands (PAI) that are present in pathogenic strains, and absent in non-pathogenic strains of one species. PAIs are genomic islands (GIs) encoding virulence factors such as toxins, adhesins or invasins. Identification of GIs is primarily based on a different G+C content from the rest of the genome and on their association with insertion sequence (IS) elements or tRNA genes at their boundaries [26], [27].

There are several identified islands and prophages in meningococci and gonococci [23], [28]–[34]. It has been recently suggested that an island composed of 22 genes in the *N. meningitidis* isolate 053442 genome (*NMCC_0592* to *NMCC_0613*) and called IHT-G (Island of Horizontally Transferred DNA-G) could be a “meningococcal pathogenicity island-like region” [20]. This island, which is adjacent to a tRNA-Pro gene, contains genes belonging to the multiple adhesin family (*maf*). Maf proteins were first described in the gonococcal strain MS11 as ligands interacting with a specific glycolipid (GgO4) [35]. Indeed, the heterologous expression of the neisserial protein in *E. coli* allows bacterial adhesion to GgO4 [35]. Since multiple genes in the gonococcus chromosome encode these proteins, they were subsequently termed “MafA adhesins”. The gene immediately downstream of *mafA*, the function of which was unknown, was termed *mafB* because both genes are organized in a putative operon.

In this study, we analyzed loci containing *maf* genes in several strains of *N. meningitidis* and *N. gonorrhoeae*. We propose here a novel uniform nomenclature of these loci. We demonstrated experimentally that *mafB* genes encode polymorphic toxins and that genes immediately downstream of *mafB* encode a specific immunity protein (MafI). Furthermore, we demonstrated that overexpression of one of the four MafB toxins of strain NEM8013 provides an advantage in competition assays.

## Results

### General features of *maf* genomic islands in pathogenic *Neisseria* species

#### A uniform nomenclature for loci containing *maf* genes

Our *in silico* analysis of the genome of 12 strains of *N. meningitidis* (Z2491, MC58, FAM18, H44/76, M04-240196, M01-240355, NZ-05/33, WUE2594, 053442, G2136, M6190 and NEM8013) and 3 strains of *N. gonorrhoeae* (FA1090, MS11 and NCCP 11945) revealed that all strains contain several loci in which genes previously designated *mafA* and *mafB* are present. To facilitate comparison of these loci, we propose the following uniform nomenclature.

**Figure 1:**
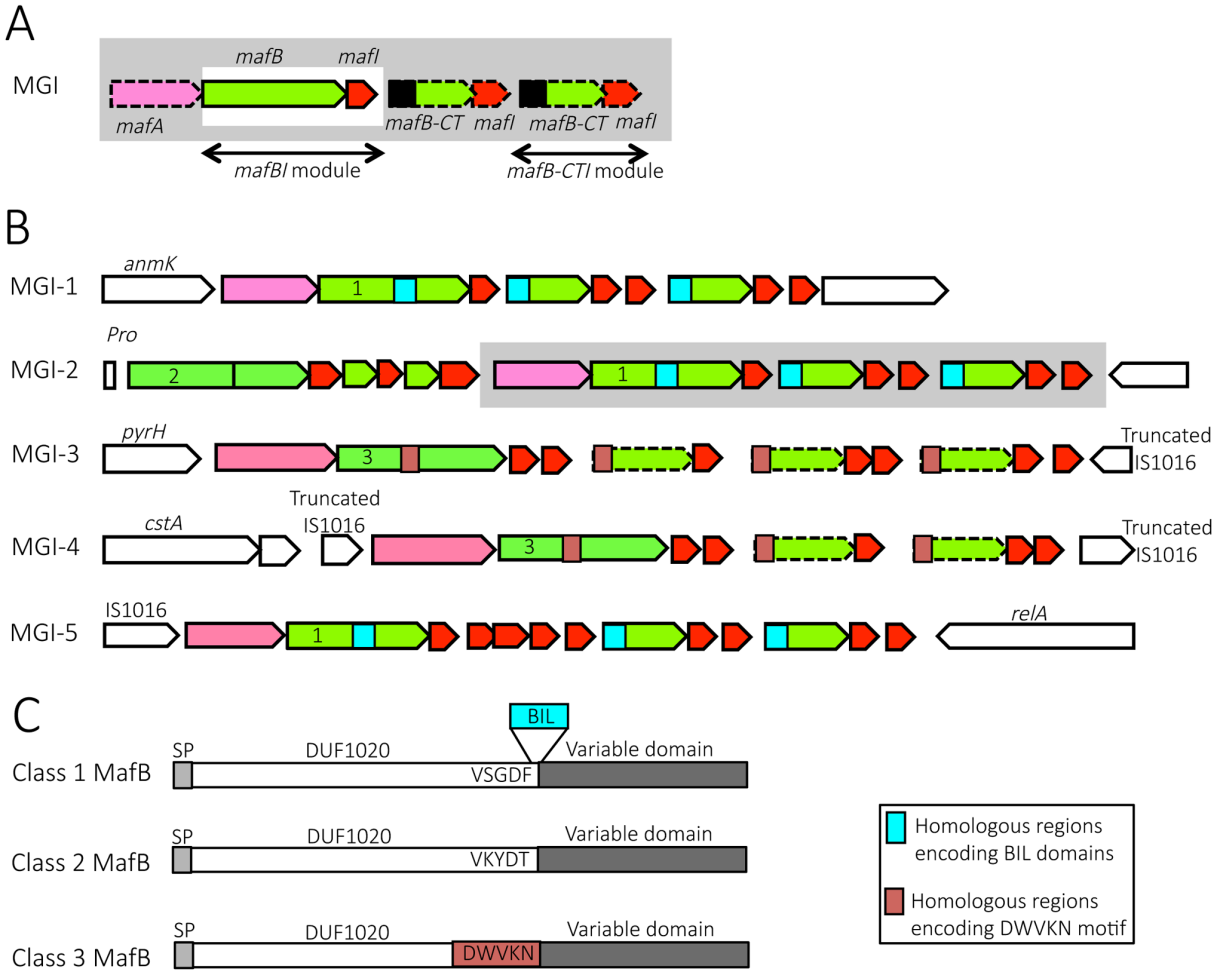
Organization and location of *maf* genomic islands in pathogenic *Neisseria* species. A) Schematic depiction of *maf* genomic island (MGI). By definition, a GI containing a *mafBI* module is a MGI. Each *mafBI* module is composed of two genes: *mafB* (green) and *mafI* (red). Additional genes (on a grey background) are *mafA* (pink), ORFs encoding alternative C-terminal domains of MafB (*mafB-CT*, green) associated with their cognate *mafI* gene (red). Black box at the 5′ end of *mafB-CT* indicates a region potentially involved in antigenic variation of MafB. In class 1 MafBs, this region encodes a bacterial intein-like (BIL) domain whereas in class 3 MafBs, this region encodes a DWVKN motif. B) Simplified genomic organizations and flanking genes of the 5 MGIs found in pathogenic *Neisseria*. MGIs 1, 2 and 3 are found in *N. meningitidis* and *N. gonorrhoeae* while MGIs 4 and 5 are only found in *N. gonorrhoeae*. MGI-1 flanking genes encode an Anhydro-N-acetylmuramic acid kinase (AnmK) on the 5′end of the island and a hypothetical periplasmic protein on the 3′end. MGI-2 flanking genes encode a Proline tRNA on the 5′end of the island and Trk system potassium uptake protein on the 3′end of the island. A cluster of genes, which is only present in some strains, is represented on a grey background. MGI-3 flanking genes encode an uridylate kinase (PyrH) and a truncated IS1016 element. MGI-4 is flanked by two truncated IS1016 elements. MGI-5 flanking genes encode an IS1016 and a ppGpp synthase (RelA). Color code: conserved flanking genes (white), *mafA* (pink), *mafB* and *mafB-CT* cassettes (green), *mafI* (red), location of sequence encoding BIL domain (blue box), location of sequence encoding DWVKN motif (salmon rose box). The number (1, 2 or 3) inside the 5′ end of *mafB* genes indicates the corresponding class of MafB (Class 1, 2 or 3 respectively). The dotted outline indicates a *mafB-CT* gene without initiation codon. C) A schematic representation of the 3 classes of MafB proteins. All MafBs contain a signal peptide (SP, light grey), a N-terminal conserved domain named DUF1020 (white) and a C-terminal variable region (dark grey). Class 1 MafBs contain a VSGDF motif at the end of the N-terminal conserved domain, and between the conserved and variable regions a bacterial intein-like (BIL) domain can be inserted (blue box). Class 2 MafBs contain a VKYDT motif at the end of the N-terminal conserved domain. Class 3 MafBs contain a DWVKN motif (salmon rose box) at the end of the N-terminal conserved domain.

**Figure 2:**
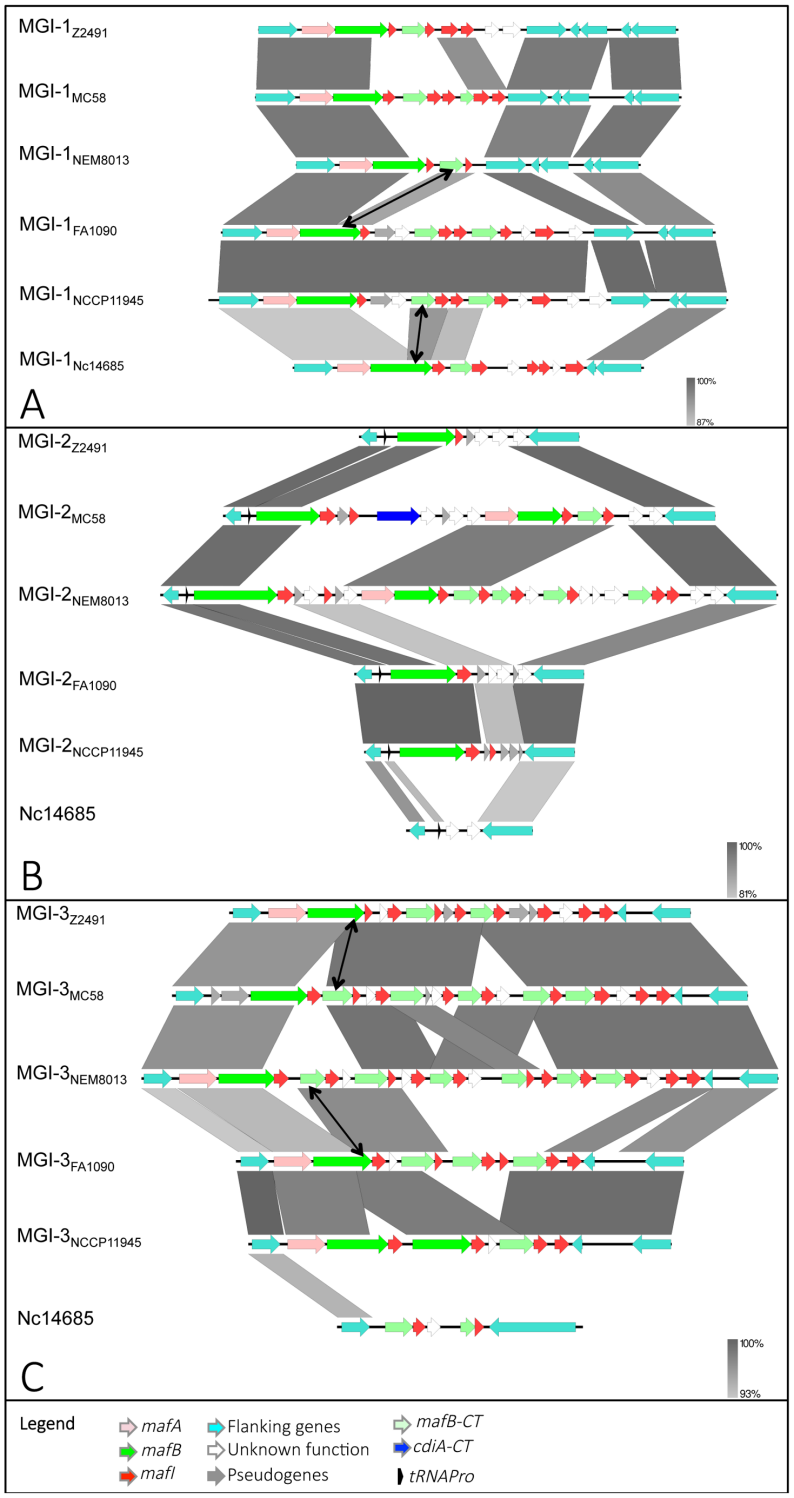
Pairwise comparison of the genetic organization of MGI-1, MGI-2 and MGI-3 loci in 6 representative *Neisseria* genomes. Nucleotide comparison of MGI-1 (A), MGI-2 (B) and MGI-3 (C) from meningococcal strains Z2491, MC58 and NEM8013, gonococcal strains FA1090 and NCCP11945 and *N. cinerea* strain ATCC 14685. Genome comparisons were generated using BLASTn implemented in Easyfig 2.1 with a cutoff value of 80%. Grey vertical blocks indicate regions of shared similarity shaded according to BLASTn identity. The level of nucleotide identity is shown in the gradient scale for each MGI. Genes are indicated with arrows colored according to their predicted functions with the color code illustrated in the legend. Double-headed arrows connect *mafB-CT* genes that are identical to the 3′ region of a full-length *mafB* gene. The orientation of genes is as in the published genomic sequences, except for MGI-1_NEM8013_, MGI-1_MC58_, MGI-2_NEM8013_, MGI-3_Z2491_ and MGI-3_FA1090_, which have been reversed for clarity purpose. Genomic regions found in *N. cinerea* at the location of MGI-2 and MGI-3 are not considered as MGIs because they do not encode a full-length MafB toxin.

Typical *maf* loci contain at least one module of two genes: *mafB* and a small ORF that we designated *mafI* (Fig. 1A). If a locus contains this module, the locus is thereafter named MGI for *maf* genomic island. MGIs have conserved chromosomal locations (Fig. 1B, Fig. 2) and exhibit hallmarks of horizontal gene transfer. They are indeed located near a tRNA gene or flanked by a transposable element (IS1016 element), and exhibit a nucleotide composition different from the rest of the genome. The average GC% of a MGI is close to 40% whereas the average GC% of a pathogenic *Neisseria* genome is close to 52%.

We propose to give the same name (MGI-1, MGI-2…) to identify different MGIs that are located at the same location across multiple strains. Since each sequenced genome exhibits a unique gene composition of MGIs, the name of the strain is mentioned as a lower index (*i.e.* MGI-1_Z2491_). We identified 5 different types of MGIs (MGI-1 to MGI-5) (Fig. 1B).

MGI-1 is located at the 3′ end of *anmK* (encoding an anhydro-N-acetylmuramic acid kinase) and at the 5′ end of a conserved gene encoding a hypothetical periplasmic protein (Fig. 1B, Fig. 2A). MGI-2 is located at the 3′ end of a tRNA-Pro gene and at the 3′ end of a conserved gene encoding a putative Trk system potassium uptake protein (Fig. 1B, Fig. 2B). MGI-3 is located at the 3′ end of *pyrH* (encoding an uridylate kinase) and at the 5′ end of a truncated IS1016 element (Fig. 1B, Fig. 2C). MGI-1, MGI-2 and MGI-3 are present in *N. meningitidis* and in *N. gonorrhoeae. N. gonorrhoeae* genomes contain two additional MGIs: i) MGI-4 is flanked by two truncated IS1016 elements that are located between a small ORF encoding a putative protein and a *nalP* pseudogene (Fig. 1B), ii) MGI-5 is located at the 3′ end of an IS1016 and at the 3′ end of *relA* (encoding a ppGpp synthase) (Fig. 1B).

To designate a gene located in a MGI, we propose to abandon the nomenclature from the initial genome annotation which was solely based on the order of appearance of *maf* genes in the genome (*i.e.* in NEM8013: *mafB1* = *NMV_0410*, *mafB2* = *NMV_1757* and *mafB3* = *NMV_2312*), and to name a gene by its nature *mafA*, *mafB* or *mafI* followed in a lower index by the name of the genomic island where it is found and the name of the strain. For example, *NMV_0410* is referred as *MafB_MGI-1NEM8013_* and *NMV_2312* is referred as *MafB_MGI-3NEM8013_*. Correspondence between locus tags, old and new nomenclature is summarized in S1 Table. If there are several *mafB* genes in a MGI, a number that refers to the position in the locus is added. This is especially the case for MGI-2 that has two *mafB* genes, the first *mafB* gene on the 5′end of the island is numbered 1. For example in MGI-2_NEM8013_, *NMV_1766* is now referred as *MafB1_MGI-2NEM8013_* and *NMV_1757* is referred as *MafB2_MGI-2NEM8013_*. The small ORF found immediately downstream of a *mafB* gene is termed *mafI, i.e. mafI1_MGI-2NEM8013_* designates the gene found immediately downstream of *mafB1_MGI-2NEM8013_*.

Strikingly, there are few or no MGIs in commensal species, in contrast to strains of pathogenic species where multiple MGIs are present. MGI-1 is found in *N. lactamica* (strain 020-06), *N. cinerea* (ATCC 14685) and *N. polysaccharea* (ATCC 43768). *N. cinerea* (ATCC 14685) and *N. polysaccharea* (ATCC 43768) do not contain additional MGI. *N. lactamica* is the non-pathogenic species that is the most closely related to the two pathogenic *Neisseria* species. In *N. lactamica* 020-06 a MGI-1 and a MGI-3 are present. A MGI-2 and a MGI-5 are also present but the corresponding *mafB* exhibit a frameshift in both of these MGIs. In the available unassembled genomes of commensal species *N. subflava* (NJ9703), *N. mucosa* (ATCC 25996 and C102 strains) or *N. elongata subsp. glycolytica* (ATCC 29315) we did not find a *mafA* gene or a full-length *mafB* gene. In *N. flavescens* (NRL 30031/H210), there is an incomplete MGI-5 with a *mafA* gene (*NEIFLAOT_01129*) and genes encoding C-terminal regions of *mafB* but without a full-length *mafB* gene.

#### Classification of MafB proteins based on their N-terminal domain

Sequence alignment of MafB proteins revealed that MafB has a N-terminal conserved domain of unknown function named DUF1020 (or PF06255 in PFAM Database) and a C-terminal (CT) variable region (Fig. 1C). DUF1020 is restricted to *Neisseria* genus and is preceded by a signal peptide domain (approximately 25 residues long). Amino acids alignment of 150 sequences of MafB proteins (see details in Materials and Methods section) revealed 3 classes of MafB with 3 conserved N-terminal regions (Fig. 1C). The percentage of sequence identity within N-terminal conserved regions of MafB proteins belonging to the same class is over 85% (90%, 97% and 89% for class 1, 2, 3, respectively) whereas the percentage of sequence identity between the conserved regions of different classes is approximately 35%.

Class 1 MafB proteins exhibit a conserved motif (VSGDF) located approximately 300 amino acids from the N-terminus (Fig. 1C and S1 Fig.). This motif is located at the transition between the conserved N-termini and the variable CT sequences. On the other hand, in class 2 and 3 MafB proteins, the conserved motifs found at the transition between the conserved and the variable regions are VKYDT and DWVKN, respectively (Fig. 1C, S2-S3 Fig.).

Some class 1 MafB proteins have a striking feature at the end of their N-terminal constant domain. Indeed, an A-type Bacterial Intein-Like (BIL) domain of approximately 140 amino acids is present at the junction between the conserved N-terminal region and the variable C-terminal region (*i.e.* in MGI-1_FA1090_) (Fig. 1C and S1 Fig.). This BIL domain is located immediately after the conserved motif (VSGDF) (S1 Fig.).

All the MafB sequences, that we analyzed using the SignalP 4.1 program [36] showed a signal peptide (SP) recognized by type 1 signal peptidase. For example in NEM8013, MafB_MGI-1NEM8013_ and MafB2_MGI-2NEM8013_ harbor a predicted cleavage site between amino acid residues 27 and 28, MafB1_MGI-2NEM8013_ between amino acid residues 24 and 25 and MafB_MGI-3NEM8013_ between amino acid residues 26 and 27. Despite the presence of two consecutive arginine residues in the signal-peptide sequence of class 1 MafBs, PRED-TAT [37], TatP 1.0 [38] or TATFIND 1.4 [39] programs did not predict a putative tat (twin arginine translocation) signal. Thus, the translocation of the 3 classes of MafB proteins through the inner membrane is likely to occur via the Sec pathway.

In most cases, the variable domain of MafB shares no homology with protein of known function. Only in few cases a homology with CT extremities of polymorphic Cdi and Rhs toxins family can be noticed. However, in contrast with Cdi and Rhs toxins no repeat domains are found in the N-terminal region of MafB.

#### Gene content of the different *maf* genomic islands

In addition to the *mafB-mafI* module, a *mafA* gene is frequently found immediately upstream of genes encoding class 1 and class 3 MafB but not upstream of genes encoding class 2 MafB (Fig. 1A, 1B). A BLASTp search using MafA sequence as a query evidenced that *mafA* is also specific of the *Neisseria* genus. The LipoP 1.0 server [40] predicts that MafA exhibits a lipoprotein signal peptide without an aspartic acid in position +2 after the cleavage site and thus could be a lipoprotein attached to the outer membrane. It has been shown by immunoelectronic microscopy [35] that MafA of gonococcal strain MS11 is surface exposed and is able to bind to glycolipids [35]. The location of MafA in the outer membrane and in outer membrane vesicles (OMV) has been confirmed by several proteomic studies [41]–[43].

Genes encoding class 1 MafB are present in MGI-1, MGI-5 and some MGI-2, whereas genes encoding class 3 MafB are present in MGI-3 and MGI-4 (Fig. 1B). Genes encoding class 2 MafB are only found in MGI-2 (Fig. 1B). Class 1 MafB encoded in the MGI-2_NEM8013_ (MafB2_MGI-2NEM8013_) and MGI-2_MC58_ (MafB2_MGI-2MC58_) differs from the class 1 MafB encoded in the MGI-1_NEM8013_ and MGI-1_MC58_ by a deletion of 50 amino acids in their N-terminal region.

Examination of *maf* clusters downstream of the full-length *mafB* gene shows many genes encoding alternative MafB-C-terminal cassettes. All these *mafB-CT* genes are followed by at least one *mafI* gene (Fig. 1A, 1B). There are two types of *mafB-CT* genes: i) *mafB-CTs* starting with an initiation codon, which are potentially translated, and ii) *mafB-CTs* devoid of initiation codon (ATG, GTG, TTG, ATT or CTG), which are potentially silent cassettes.

Intriguingly, potentially expressed cassettes are only found in MGIs encoding class 1 MafB whereas silent cassettes are only found in MGIs encoding class 3 MafB (Fig. 1B). In MGIs encoding class 1 MafB, the *mafB-CTs* that have an initiation codon encode an A-type BIL (Fig. 1B) and have no homologous region with the conserved region of the full-length class 1 MafB (Fig. 1B) that could allow a recombination. The initiation codon for the translation of the BILs is a TTG and is the initiation codon of the *mafB-CT*. It should be pointed out that BILs of the MafB-CTs lack the first 7 amino acids compared to complete BIL sequence. On the other hand, the full-length *mafB* genes located upstream of these *mafB-CTs* may contain a complete BIL sequence (*i.e.* in MGI-1_FAM18_).

In the MGI containing class 3 MafB, the *mafB-CTs* are gene fragments that usually encode a conserved WDWVKN motif present in the full-length class 3 MafB, but as mentioned above these *mafB-CTs* do not have an initiation codon (Fig. 1B).

The *maf* clusters harbor numerous small ORFs, designated *mafI*, immediately downstream of *mafB*. Similarly to the high variability of MafB-CT, MafI sequences are also highly variable, suggesting that the encoded proteins could specifically interact with cognate MafB-CTs. In support of this hypothesis, when two *mafB-CTs* are almost identical their associated *mafI* gene are also identical. For instance, the C-terminal region of MafB_MGI-3Z2491_ (NMA0324) and a cassette (NMB2107) found in MGI-3 of MC58 exhibit 100% amino acid identity (Fig. 2C). The corresponding immunity proteins MafI_MGI-3Z2491_ (NMA0323) and NMB2108 are also 100% identical (Fig. 2C). Similarly, MafB_MGI-3FA1090_ (NGO1971) and a cassette (NMV_2314) found in MGI-3 of NEM8013 exhibit 90% amino acid identity (Fig. 2C). The corresponding immunity proteins MafI_MGI-3FA1090_ (NGO1970) and NMV_2315 are 100% identical (Fig. 2C).

A search in the Conserved Domain Database (CDD) [44] of the NCBI revealed that many *mafI* genes encode proteins containing domains typically found in putative immunity proteins of bacterial polymorphic toxin systems [1], [2]. These recently described domains include Imm17, Imm21, Imm22, Imm47, SUFU and SMI1 domains. However, almost half of the small ORFs in MGIs do not contain any known domains. Small ORFs are considered immunity genes if they are located immediately downstream of a *mafB-CT* gene or if the corresponding amino acids sequence contain a predicted immunity domain in CD search database.

### *mafB-mafI* modules encode a new family of toxin-immunity

Analysis of the amino acid sequences of several MafB-CT regions using the CDD server revealed homologies with putative or known toxic domains. For instance, proteins encoded by *mafB_MGI-1NEM8013_* (*NMV_0410*) contains a domain belonging to the RNase EndoU-fold, *mafB_MGI-1FA19_* (*NGEG_01276*) contains a domain belonging to a nucleotide deaminase superfamily and *mafB_MGI-5FA1090_* (*NGO1392*) contains a domain belonging to the DNase HNH/EndoVII-fold. In this study, we decided to focus on the four putative MafB toxins encoded in meningococcal strain NEM8013 which are MafB_MGI-1NEM8013_, MafB2_MGI-2NEM8013_, MafB_MGI-3NEM8013_ and MafB1_MGI-2NEM8013_ (formerly MafB1, MafB2, MafB3 and MafB-related respectively).

#### MafB_MGI-1NEM8013_ and MafB _MGI-3NEM8013_ proteins of strain NEM8013 inhibit cell growth when expressed in *E. coli* and expression of cognate MafI counteracts MafB toxicity

**Figure 3:**
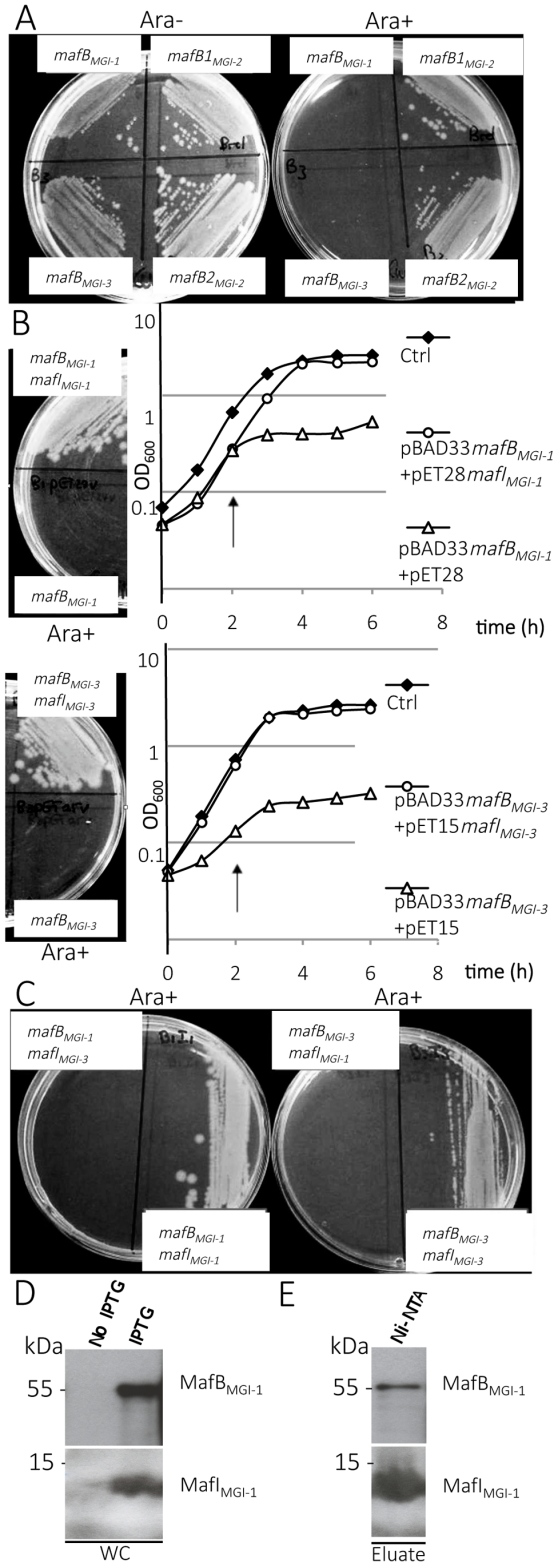
Toxic effect of MafB proteins of NEM8013 strain on *E. coli* growth. A) Effect of NEM8013 MafB putative toxins over-expression on *E. coli* grown in the presence of 0.2% L-arabinose (Ara+) or without arabinose (Ara−). BL21(DE3) cells were transformed with vector pBAD33 carrying *mafB* genes found in NEM8013 strain (*mafB_MGI-1NEM8013_, mafB1_MGI-2NEM8013_, mafB2_MGI-2NEM8013_* and *mafB_MGI-3NEM8013_*). B) Inhibition of growth due to the toxin is counteracted by cognate immunity protein co-expression on LB agar plates (0.2% L-arabinose) and in LB broth. LB agar plates and LB broth contain 0.01 mM IPTG to induce expression of *mafI*. Toxin expression was induced by adding 0.2% L-arabinose in LB broth 2 h after inoculation (arrow). *mafB_MGI-1NEM8013_* and *mafB_MGI-3NEM8013_* are cloned in pBAD33, *mafI_MGI-1NEM8013_* is cloned in pET28 and *mafI_MGI-3NEM8013_* is cloned in pET15. Control strains (Ctrl) contain empty vectors. C) Co-expression of a non-cognate immunity protein does not confer protection against MafB toxicity. LB agar plates contain 0.01 mM IPTG and 0.2% L-arabinose. The results shown are from one of three independent experiments. D) MafB_MGI-1NEM8013_ and MafI_MGI-1NEM8013_ copurify. *mafB* and *mafI* from MGI-1_NEM8013_ were cloned under the control of two independent T7 promoters in plasmid pcolaDUET. Upon induction with IPTG, *E. coli* BL21(DE3) carrying pcolaDuet-*mafBmafI* expressed His_6_- MafI_MGI-1NEM8013_ and MafB_MGI-1NEM8013_, as evidenced by immunoblotting of whole-cell lysates (WC). E) His_6_-tagged MafI_MGI-1NEM8013_ and MafB_MGI-1NEM8013_ were recovered from Ni-NTA-agarose column eluate (Eluate). Copurified proteins were analysed by immunoblotting. Antibodies used for immunoblotting of WC and Ni-NTA eluate were Anti- MafB_MGI-1NEM8013_ and Anti- MafI_MGI-1NEM8013_. Ni-NTA, Nickel-nitrilotriacetic acid.

To assess the putative function of *mafB-mafI* modules, we expressed the four predicted MafB toxins of strain NEM8013 in *E. coli*. We individually cloned the four *mafB* genes of strain NEM8013 into tightly controllable expression vector pBAD33 [45]. Induction of the expression of *mafB_MGI-1NEM8013_* and *mafB_MGI-3NEM8013_* was highly toxic for *E. coli* both on agar Luria-Bertani (LB) plates and in liquid LB culture (Fig. 3). Induction of the expression of *mafB_MGI-1NEM8013_* and *mafB_MGI-3NEM8013_* resulted in growth inhibition. On the other hand, induction of *mafB1_MGI-2NEM8013_* and *mafB2_MGI-2NEM8013_* genes did not alter growth of *E. coli* (Fig. 3A). Because some toxins targets are only present in the periplasmic space, we cloned *mafB1_MGI-2NEM8013_* and *mafB2_MGI-2NEM8013_* devoid of their own signal sequence in pET-22 and added *pelB*-signal sequence at the N-termini of both proteins to direct them to *E. coli* periplasm. MafB1_MGI-2NEM8013_ and MafB2_MGI-2NEM8013_ remained non-toxic even when exported to *E. coli* periplasm (S4A Fig.). These results demonstrate that a toxic activity could be detected only for MafB_MGI-1NEM8013_ and MafB_MGI-3NEM8013_.

In order to test whether the toxicity of MafB resides in its C-terminal domain, we cloned only the DNA region encoding the C-terminal domain or the-N terminal domain of MafB_MGI-1NEM8013_ in *E. coli* using pBAD33. As expected, expression of the C-terminal domain of MafB_MGI-1NEM8013_ was toxic for *E. coli* but expression of the N-terminal domain had no effect on *E. coli* growth (S4B–S4C Fig.).

To verify if the small ORF immediately downstream of the *mafB* gene was indeed an immunity gene, we cloned *mafI_MGI-1NEM8013_* and *mafI_MGI-3NEM8013_* under the control of the IPTG inducible promoter of pET-28 and pET-15 respectively. While the expression of MafB_MGI-1NEM8013_ or MafB_MGI-3NEM8013_ inhibited growth of *E. coli*, the co-expression of MafB_MGI-1NEM8013_ and MafI_MGI-1NEM8013_ or the co-expression of MafB_MGI-3NEM8013_ and MafI_MGI-3NEM8013_ did not impede growth of *E. coli* (Fig. 3B). There was no cross-protection conferred by MafI_MGI-1NEM8013_ against MafB_MGI-3NEM8013_ or by MafI_MGI-1NEM8013_ against MafB_MGI-3NEM8013_ (Fig. 3C). Thus, the small ORF adjacent to the toxin *mafB* gene encodes a protein providing immunity to the cognate toxin and preventing self-intoxication.

To explore the mechanism of toxin inactivation we investigated a potential direct toxin-antitoxin binding. For this, we cloned in the same vector (pcolaDUET) under two different IPTG promoters, *mafB_MGI-1NEM8013_* and *mafI_MGI-1NEM8013_* in order to co-express in *E. coli* both proteins. Only MafI_MGI-1NEM8013_ harbored a His_6_-tag. We tested whether MafB_MGI-1NEM8013_ co-purified with His_6_-tagged MafI_MGI-1NEM8013_ using Ni^2+^-affinity chromatography. Despite the very low expression of the MafB_MGI-1NEM8013_ toxin in *E. coli*, we could co-purify MafB_MGI-1NEM8013_ with His_6_-tagged MafI_MGI-1NEM8013_ (Fig. 3D–E). Thus MafI_MGI-1NEM8013_ is likely to inhibit MafB_MGI-1NEM8013_ toxicity by a direct interaction.

#### MafB_MGI-1NEM8013_, MafB_MGI-3NEM8013_ and MafB1_MGI-2NEM8013_ proteins are toxic in their original NEM8013 *Neisseria* strain

In a first attempt to assess the toxicity of MafB proteins in meningococcus, we tried to generate mutants of the immunity gene located downstream of each of the four *mafB* genes in NEM8013. It was impossible to delete the immunity gene of *mafB_MGI-1NEM8013_*, *mafB1_MGI-2NEM8013_* and *mafB_MGI-3NEM8013_* (respectively *mafI_MGI-1NEM8013_*, *mafI1_MGI-2NEM8013_* and *mafI_MGI-3NEM8013_*). Every attempt of mutagenesis led to duplication of the gene. On the other hand, the replacement of *mafI2_MGI-2NEM8013_* immunity gene by a chloramphenicol resistance cassette was possible. We next tried to insert an ectopic copy of the four *mafB* genes in the NEM8013 genome using pGCC4 constructs (see Materials and Methods section) [46]. Similar results as above were obtained and no strain carrying an additional copy of *mafB_MGI-1NEM8013_*, *mafB1_MGI-2NEM8013_* and *mafB_MGI-3NEM8013_* could be obtained. On the other hand, it was possible to insert an additional copy of *mafB2_MGI-2NEM8013_* in the wild-type (WT) strain and in a *mafI2_MGI-2NEM8013_* deleted mutant. On the other hand, it was possible to insert an additional ectopic copy of the four whole modules (*mafB* with *mafI*) using pGCC4 constructs. These results evidenced that all MafB proteins of strain 8013 except MafB2_MGI-2NEM8013_ have a toxic effect in NEM8013, unlike what was observed in *E. coli* where MafB1_MGI-2NEM8013_ and MafB2_MGI-2NEM8013_ were non-toxic.

#### Overexpression of the toxin MafB1_MGI-2NEM8013_ gives a competitive advantage

**Figure 4:**
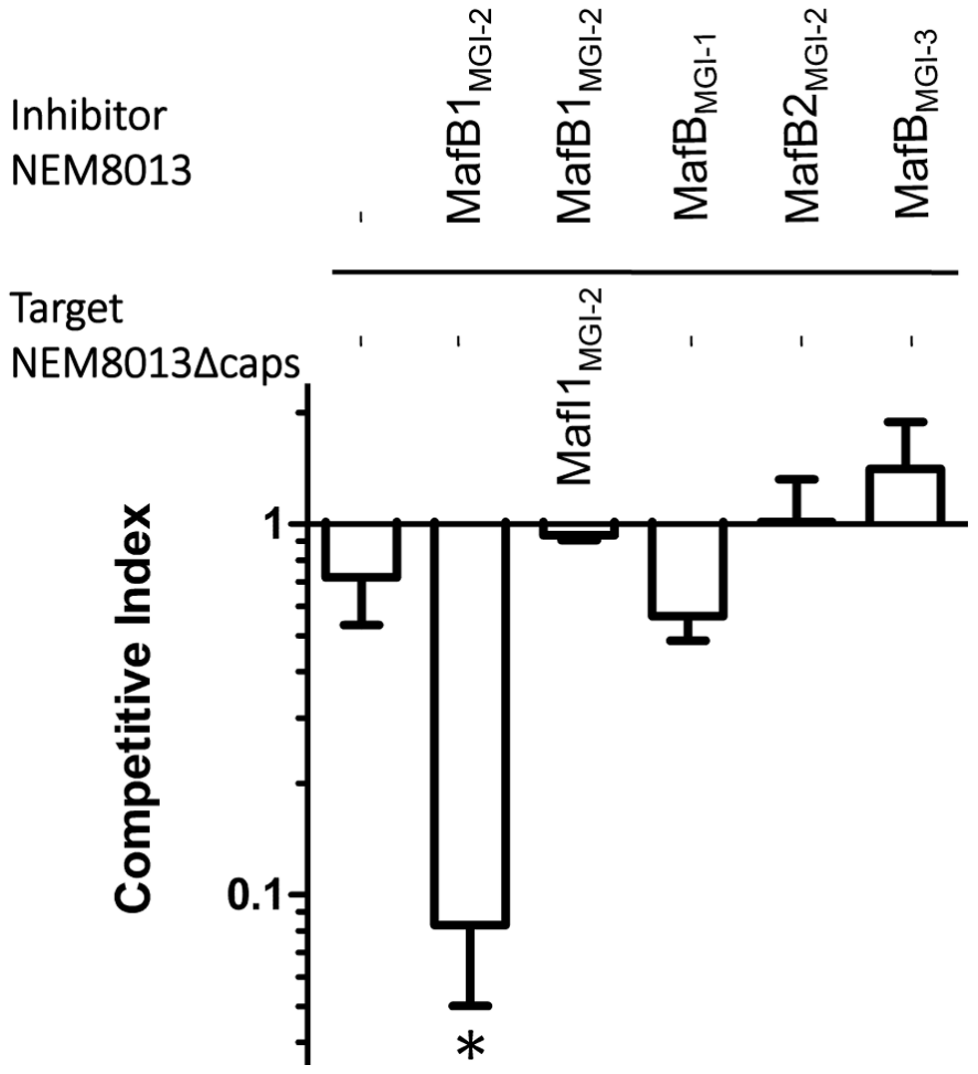
MafB1_MGI-2NEM8013_ provides an advantage in competition assay. Competition assays were performed with an initial inhibitor to target cell ratio of 10 to 1. Putative inhibitory cells were NEM8013 overexpressing each of the four *mafB* toxins and their cognate *mafI* immunity genes using pGCC4 constructs. Putative target cells were an unencapsulated derivative of NEM8013 with a transposon insertion in *ctrA* gene (NEM8013Δcaps) or this unencapsulated derivative overexpressing MafI1_MGI-2NEM8013_. The overexpressed toxin of the inhibitor cells is indicated (otherwise a – indicates that there is no overexpressed toxin) and the overexpressed immunity of the target cells is indicated (otherwise a – indicates that there is no overexpressed immunity protein). Mixed cultures were spotted on a membrane filter placed on GCB agar plate containing 1 mM IPTG and incubated overnight. Filters recovered after overnight incubation were used to perform viable counts and the competitive index was calculated as the inhibitor/target ratio in the output divided by the initial inhibitor/target ratio. The data from three independent experiments were examined for significance using a two-tailed Student's t-test. * *p*-value *p*<*0.05*.

Since Cdi and Rhs toxins have been involved in inter-bacterial competition, we searched for a role of MafB in competition assays. We used strain NEM8013 and tested the impact of the overexpression of the four MafB proteins in competition assays. Constructions overexpressing each of the four MafB toxins with their cognate MafI proteins, in strain NEM8013, were used as inhibitor cells. As it has been shown previously that expression of a capsule could block CdiA mediated toxicity [47], we used an unencapsulated derivative of meningococcal strain NEM8013 as target cell. Competition assays were performed overnight on a solid media at an inhibitor to target ratio of 10∶1 (see Materials and Methods). Under these experimental conditions, we evidenced a significant competitive advantage only for the strain overexpressing MafB1_MGI-2NEM8013_ (Fig. 4). The advantage was lost when target cells overexpressed the cognate immunity MafI1_MGI-2NEM8013_ (Fig. 4). These data suggest that MafB1_MGI-2NEM8013_ could be employed by strain NEM8013 to outcompete strains that do not possess or express at a sufficient level the cognate immunity. Besides, the fact that the introduction of an additional copy of *mafB1_MGI-2NEM8013_* in wild-type NEM8013 is impossible without the simultaneous introduction of an additional copy of the cognate immunity gene, suggests that the toxicity resulting of an overexpression of the toxin cannot be counteracted by the basal expression of the cognate immunity. Altogether these data demonstrate that MafB1_MGI-2NEM8013_ has a toxic effect on a non-capsulated derivative of strain NEM8013.

#### MafB toxins are secreted in a signal-peptide dependent manner

**Figure 5:**
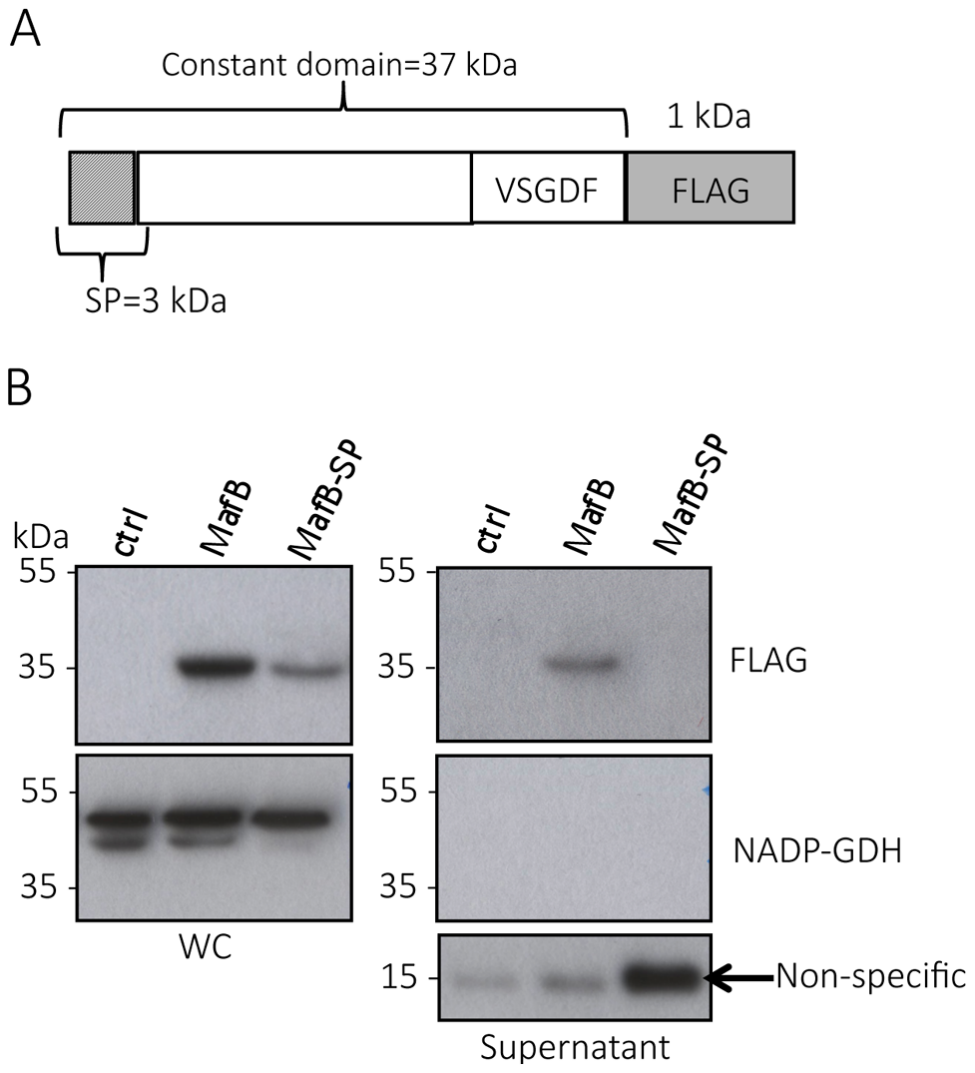
MafB_MGI-1NEM8013_ is a secreted toxin. A) Schematic representation of the construct expressed under IPTG inducible promoter in NEM8013. This construction has been inserted in an intergenic region of NEM8013 chromosome using pGCC4 vector. B) C-terminal FLAG-tagged MafB_MGI-1NEM8013_ (MafB) with or without its signal sequence (MafB-SP) are detected in the whole-cell lysates (WC) of NEM8013 strain expressing MafB or MafB-SP under an IPTG inducible promoter. NEM8013 parental strain is used as a control (ctrl). MafB is detected in the supernatant only when its signal sequence is present. As the production of MafB-SP was less efficient than the production of MafB, a larger quantity of supernatant was loaded as shown by the intensity of the nonspecific band (arrow) detected with Anti-FLAG antibody. Antibodies used for immunoblotting of WC and supernatants were Anti-NADP-GDH (NADP-dependent glutamate dehydrogenase, as a cytoplasmic marker protein) and Anti-FLAG to detect C-terminal, FLAG-tagged MafB.

We focused on MafB_MGI-1NEM8013_ (Class 1 MafB) and MafB_MGI-3NEM8013_ (Class 3 MafB) of strain NEM8013 and we engineered NEM8013 strains chromosomally expressing a FLAG-tagged version of these proteins (Fig. 5A). Using Western blotting, we were able to detect MafB_MGI-1NEM8013_ (Fig. 5B) in the culture supernatant. MafB_MGI-1NEM8013_ was no longer detected in the supernatant of a strain expressing MafB_MGI-1NEM8013_ devoid of its signal peptide (Fig. 5B). Similar results were obtained after overexpression of the wild type sequence of MafB_MGI-1NEM8013_ instead of the FLAG-tagged version using anti MafB_MGI-1NEM8013_ antibodies (S5 Fig.). Thus, the first step of MafB secretion across the inner membrane involves signal peptide recognized by the general secretory pathway. To gain access to the culture medium MafB has to cross the outer membrane, potentially through an outer membrane secretin. As PilQ is the only characterized secretin in *Neisseria* sp, we overexpressed MafB_MGI-1NEM8013_ construction in a *pilQ* mutant of strain NEM8013. The lack of PilQ secretin had no impact on MafB_MGI-1NEM8013_ level in the culture supernatant (S5 Fig.). We also aimed at studying the impact of MafA on MafB secretion. We used *N. cinerea* ATCC 14685 that possesses only one copy of *mafA*. Deletion of the unique copy of *mafA* in *N. cinerea* did not prevent secretion of MafB-FLAG when the strain was transformed with *pGCC4-mafB_MGI-1NEM8013_FLAG* (S6A–S6B Fig.). Besides, we could not evidence the presence of MafB_MGI-1NEM8013_ in *E. coli* supernatant when the protein was produced from a pET vector (S6C Fig.). Taken together, these data suggest that secretion of MafB requires a Neisseria-specific factor that is neither PilQ nor MafA.

#### MafB_MGI-1NEM8013_ is a bacterial EndoU nuclease that can degrade multiple sources of RNA

**Figure 6:**
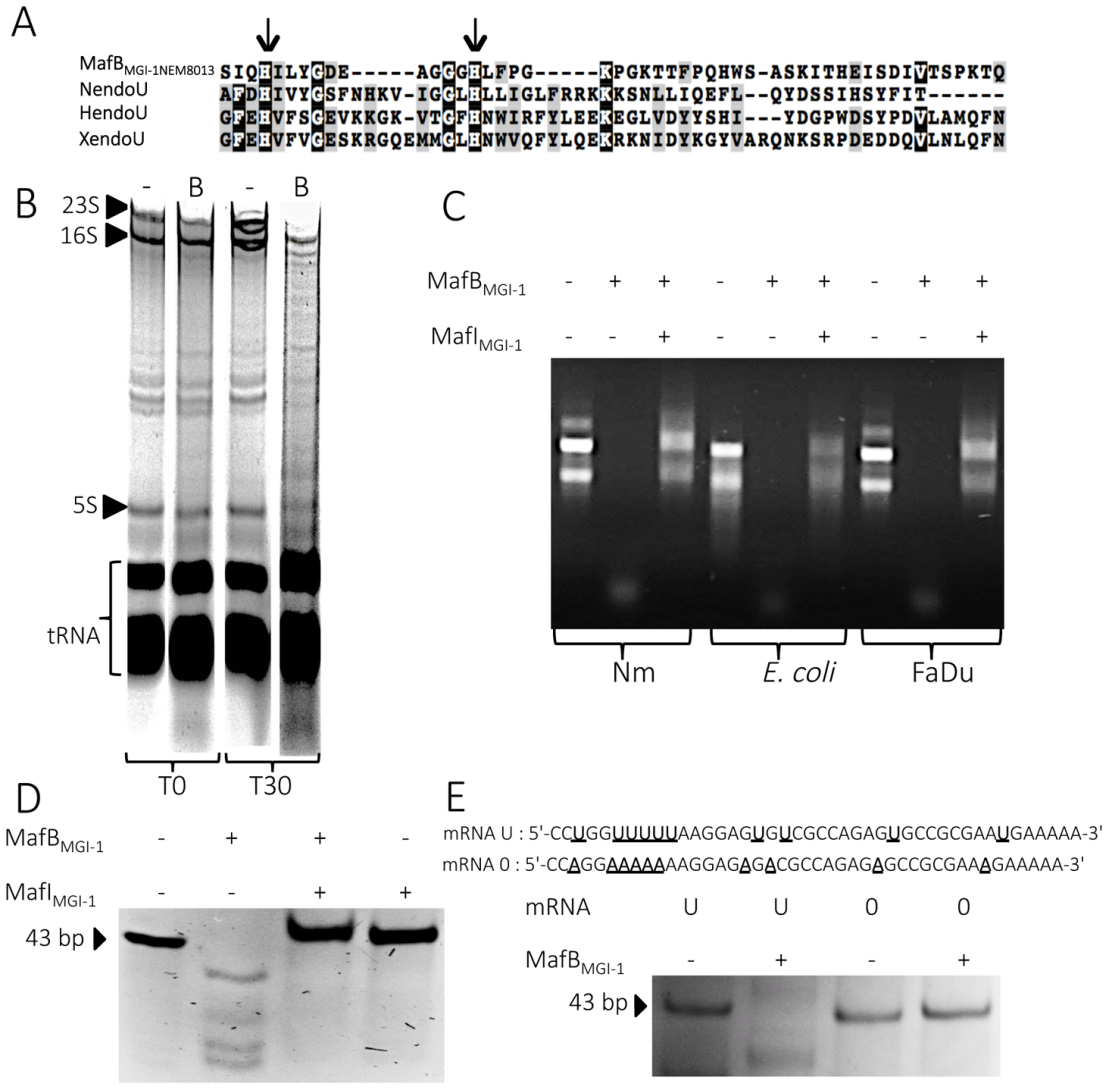
MafB_MGI-1NEM8013_ is a bacterial EndoU nuclease. A) Partial sequence alignment of EndoU nuclease domain of Nidovirus Nsp15 protein (NendoU; NP_740619), *Xenopus laevis* XendoU (Q8JFY9), human placental protein PP11 (HendoU P21128) and MafB_MGI-1_ from NEM8013 strain (C9X2Z7). The arrows indicate two conserved histidine residues, which are part of the catalytic site of previously characterized EndoU nucleases. Multiple alignment was performed using MUSCLE and shaded using the BoxShade server. Residues that are identical or similar in at least three of the four sequences are shaded with black or grey background respectively. B) Analysis of *in vivo* impact of MafB_MGI-1NEM8013_ expression in *E. coli*. Total RNA from *E. coli* expressing MafB_MGI-1NEM8013_ (pBAD33-*mafB_MGI-1NEM8013_*) was isolated before induction (T0) and 30 min after addition of L-arabinose (T30). Samples were run on 5% denaturing polyacrylamide gels and stained with ethidium bromide. Positions of 23S, 16S, and 5S rRNAs and tRNAs are shown. -, empty vector control; B, *E. coli* expressing MafB_MGI-1NEM8013_ from pBAD33 C) RNase activity of purified recombinant MafB_MGI-1NEM8013_ was assessed by incubating MafB_MGI-1NEM8013_ -His_6_ alone or with MafI_MGI-1NEM8013_ -His_6_ with total RNA isolated from different sources (*N. meningitidis* NEM8013, *E. coli* TOP10 and human epithelial cells FaDu). Each reaction was performed for 30 min at 37°C with 4 µg of RNA in Tris-EDTA buffer. D) Synthetic mRNA of 43 bp was incubated with purified MafB_MGI-1NEM8013_-His_6_ alone or with MafI_MGI-1NEM8013_ -His_6_ in Tris-EDTA buffer for 15 min at 37°C. The cleavage products were separated by electrophoresis in a 14% polyacrylamide/8M urea gel and were visualized by ethidium bromide staining. E) Synthetic oligoribonucleotides containing several uridylates (U) or none (0) were incubated with purified MafB_MGI-1NEM8013_-His_6_ in Tris-EDTA buffer for 20 min at 37°C. The reaction products were analyzed by 14% polyacrylamide/8 M urea gel. Sequences of synthetic oligonucleotides used in this experiment are shown.

Previous bioinformatics studies conducted by Zhang et coll. (in Supplementary material of [2]) suggested that MafB2_MGI-2Nm053442_ (encoded by gene *NMCC_0602* GI: 161869586) could contain a nucleasic toxin domain of the EndoU fold. Amino acid sequences comparison between MafB_MGI-1NEM8013_ and MafB2_MGI-2Nm053442_ showed 95% identity over the entire length and 100% identity over the CT region. A search in the GenBank database with the BLASTp program using the CT domain of MafB_MGI-1NEM8013_ protein as a query confirmed the presence of a putative EndoU catalytic domain with two conserved histidine residues (Fig. 6A).

First, we examined total RNA from *E. coli* cells expressing MafB_MGI-1NEM8013_ for evidence of RNA degradation. Gel analysis of total RNA isolated from cells before and after induction of MafB_MGI-1NEM8013_ production revealed that expression of MafB_MGI-1NEM8013_ led to the non-specific cleavage of most cellular RNAs (Fig. 6B). To further characterize MafB_MGI-1NEM8013_ ribonuclease activity, we purified a recombinant His_6_-tagged- MafB_MGI-1NEM8013_ protein and incubated it with various nucleic acids substrates. Recombinant MafB_MGI-1NEM8013_ was able to degrade purified total RNA from *E. coli*, *N. meningitidis* and human epithelial cells (Fig. 6C). Thus, MafB_MGI-1NEM8013_ is a ribonuclease. Incubation of MafB_MGI-1NEM8013_ with a synthetic RNA confirmed the RNAse activity (Fig. 6D). Addition of MafI_MGI-1NEM8013_ blocked the toxic activity of MafB_MGI-1NEM8013_ towards RNA (Fig. 6D). In order to determine whether MafB_MGI-1NEM8013_ ribonuclease had an uridylate specific activity, characteristic of the EndoU fold, we obtained a similar synthetic RNA where all the Us were replaced by As. This synthetic RNA was no longer cleaved even when incubation was extended up to 1 hour (Fig. 6E). Our results evidenced that MafB_MGI-1NEM8013_ preferred cleavage sites contain uridylates. Thus, with MafB_MGI-1NEM8013_, we characterized the first bacterial EndoU ribonuclease.

## Discussion

In this study, we have demonstrated that *mafB* encodes a functional polymorphic toxin and *mafI*, the downstream gene, a specific immunity protein. Focusing on MafB_MGI-1NEM8013_, we were able to characterize its EndoU ribonuclease activity. Toxins carrying EndoU activities are predicted to be widespread among diverse polymorphic toxin systems, however no EndoU activity carrying toxins had been previously experimentally confirmed in bacteria.

Polymorphic toxins encompass numerous families distributed in all bacterial lineages. Nevertheless, very few of these systems have been experimentally characterized except the Cdi and the Rhs families in Gram-negative bacteria [6], [8] and the PF04740 family from Gram-positive bacteria [48]. CdiA and Rhs toxins are the best characterized and can be found in many species including *E. coli, Yersinia pestis, Dickeya dadantii* or *Burkholderia pseudomallei* [8]. CdiA and Rhs toxins are large filamentous proteins (over 1000 amino acids) with multiple repetitive elements. In contrast, MafB toxins are predicted to have a globular structure. Organization of the loci encoding CdiA and Rhs toxins shares similarities with MGIs organization. In particular, the presence of genes encoding CT cassettes/immunity modules downstream of the toxin gene is a common feature [8]. Interestingly, *mafB* genes are restricted to the genus *Neisseria*, which is very unusual in polymorphic toxin systems [1]. A *mafA* gene, encoding a surface exposed outer membrane lipoprotein, is frequently found immediately upstream of *mafB*. *mafA* is also specific of the *Neisseria* genus. It has been shown that gonococcal MafA binds to glycolipids but its biological function remains elusive [35]. Unlike CDI two-partner secretion system in which CdiB mediates the secretion of CdiA [49], the *mafA* gene located 5′ of *mafB* shares no homology with known secretion systems. Furthermore, the sequence of MafA offers no other clues to its function.

As observed for *maf* genes, there are several loci bearing haemagglutinin related genes in meningococcal genomes. Haemagglutinin related genes are termed *tpsA* and *tpsB* genes. *tpsA* encodes a secreted filamentous haemagglutinin and *tpsB* encodes its dedicated transporter. Of note, haemagglutinin related genes are only present as pseudogenes in gonococcal genomes [50]. Analysis of the sequences of the TpsA proteins encoded in *N. meningitidis* genomes revealed the presence of three distinct groups [51]. Group 1 is found in all meningococcal genomes whereas *tpsA* genes of group 2 and 3 are overrepresented in disease isolates compared to carriage isolates [51]. In meningococcus, the number of *tpsA* genes range from 1 to 5 [52]. Genomes of strains FAM18, B16B6 and Z2491 contain only one *tpsA* gene of group 1 system while strain MC58 contains five *tpsA* genes (*NMB0493, NMB0497, NMB1214, NMB1768* and *NMB1779*) belonging to the three different groups [51].

The *tps* locus of meningococcal strain FAM18 is referred as a *cdi* locus in the comparative genomic study conducted by Poole *et al.* in 2011 [4]. Indeed, the FAM18 *tpsA* gene (*NMC0444* also termed *cdiA* by Poole *et al.*) encodes a protein that exhibits a filamentous haemagglutinin family N-terminal domain and a Pre-toxin domain with a VENN motif, which is located before the putative C-terminal toxin domain. Since this study, several recent works have been published on the meningococcal *tps* loci. It has been shown that meningococcal strain B16B6 had an advantage in competition assay against a B16B6 deletion mutant lacking the cognate immunity gene of *tpsA* [14]. The *tpsA* gene of strain B16B6 (HQ420265) encodes a protein with >99% sequence identity with that of FAM18 [14] confirming the prediction of Poole *et al.* [4] that meningococcal TpsA proteins from group 1 constitute functional CDI systems. It remains to be investigated whether TpsA proteins from group 2 and 3 are also able to mediate growth inhibition.

The six secretion pathways identified in Gram-negative bacteria can be classified in two categories, either the pathways transporting proteins in a single-step across both inner and outer membranes (*i.e.* type I, III, IV and VI) or the two-step secretion pathways (*i.e.* type II and V), where proteins are first targeted to a machinery that recognizes their N-terminal signal peptide [53]–[55]. Type V secretion systems encompass auto-transporter proteins (type Va) and two-partner secretion systems (type Vb) [54]. Cdi toxins belong to the type Vb subclass. Only type I, Va and Vb secretion pathways are found in *N. meningitidis*, whereas only type IV and Va secretion pathways are found in *N. gonorrhoeae* [56]. Thus, the only common secretion pathway shared by pathogenic *Neisseria* species is autotransporters pathway. Autotransporters are single polypeptides that consist of a surface-exposed variable N-terminal domain (“passenger domain”) that can be released from the cell surface by a proteolytic cleavage and a C-terminal domain (“translocator domain”) folded into a β-barrel structure in the outer membrane. The β-barrel of translocator domains is in most cases composed of 14 β-strands [57]. The IgA protease of *N. gonorrhoeae* was the first described autotransporter [58]. Since MafB toxins possess an N-terminal signal peptide and are found in *N. meningitidis* and *N. gonorrhoeae*, they are likely to be secreted by a two-step secretion pathway found in both species and not yet identified. Indeed, according to their domains organization and the lack of β-barrel structure prediction (using TBBpred server [59] and HMM-TM server [60]), MafB toxins do not belong to the autotransporters family. Besides, secreted proteins known to be targeted to a TpsB transporter of two-partners systems exhibit a TPS secretion domain adjacent to the signal peptide sequence [61]. There is no such TPS domain in the N-terminal region of MafBs that could target the toxin to a TpsB transporter. Thus, in contrast to Cdi toxins, MafB toxins are unlikely to be secreted via type V secretion systems. Moreover, in contrast to Rhs toxins that can be secreted through type VI secretion system, there is no type VI secretion system in pathogenic *Neisseria* species.

In addition to these six types of secretion systems, Gram-negative bacteria, including *Neisseria* pathogenic species, constitutively produce outer membrane vesicles (OMV) during their normal growth [62], [63]. OMVs are mainly composed of outer membrane and periplasmic components. OMVs enable the secretion of virulence factors to the surrounding environment or directly to neighboring bacteria [64], [65] or to eukaryotic cells [66], [67]. For instance, OMVs derived from *P. aeruginosa* is able to kill other bacterial species [64] by the release of murein hydrolases capable of degrading the peptidoglycan of other species. *P. aeruginosa* is also able to deliver multiple virulence factors directly into the host cell cytoplasm by fusion of OMV with host cell membrane lipid raft [67]. Thus, OMVs can interact both with competing bacteria and with host cell to promote bacterial colonization of the host or pathogenesis. Pathogenic *Neisseria* are well known for their release of OMVs [56], [62]. Natural and engineered OMVs have recently gained interest for use as vaccine or adjuvants. The OMV vaccine strategy has been successfully used during several clonal outbreaks of serogroup B meningococcal strains in Cuba, Norway, and New Zealand [68]. As a consequence, several recent proteomic studies analyzed the *Neisseria* OMVs content. A proteomic study of naturally released OMVs isolated from four gonococcal strains (FA1090, F62, MS11, and 1291) revealed the presence of MafA and MafB_MGI-2_ (corresponding to ORFs *NGO0225, NGNG_00563, NGFG_00362* and *NGAG_00430*) in OMVs of 4 strains [43]. In addition, supplemental data published by Zielke *et al.* in the same proteomic study suggest that four other MafB toxins produced by these gonococcal strains could also be present in OMVs. Since MafA has been previously described as an adhesin able to bind cellular glycolipids [35], the presence of MafA in OMVs could mediate attachment of OMVs to eukaryotic cells. This suggests that OMV-mediated release could be a mean for delivery of MafB toxins to neighboring bacteria or to eukaryotic cells. The presence of MafB toxins in OMVs and its potential implications for *Neisseria* pathogenesis have to be further explored.

A hallmark of MGIs is the presence of numerous *mafB-CT* genes. Genetic recombination, resulting from the replacement of the 5′ end of a full-length *mafB* gene by an alternative CT cassette is supported by genome comparison both for MGI encoding class 1 or class 3 MafBs. For example in MGI-1s, the *mafB*-*CT* cassette found in MGI-1_NEM8013_ (*NVM_0408*) encodes the same CT region that *mafB_MGI-1FA1090_* (Fig. 2A). In MGI-3s, the *mafB*-*CT* cassette found in MGI-3_NEM8013_ (*NVM_2314*) encodes the same CT region that *mafB*_MGI-3FA1090_ (Fig. 2C). Genetic recombination of polymorphic toxins has been recently demonstrated for CdiA in *N. meningitidis* [14] and for Rhs in *Salmonella enterica* serovar Typhimurium [10].

In this study, we demonstrated a functional role for one of the four MafB toxins of NEM8013. Indeed, we showed that an unencapsulated derivative of NEM8013 was outcompeted by a strain overexpressing MafB1_MGI-2NEM8013_. Several studies have evidenced that meningococci frequently become acapsulated in the nasopharynx as a result of phase variation [69], [70] or by down-regulation of the genes involved in capsule biosynthesis [71], [72]. Thus, both capsulated and unencapsulated strains are likely to compete with each other for colonization of the nasopharyngeal niche. The function of the three other *mafB* genes of this strain remains unknown. Given the diversity of the CT extremities of MafBs, it is also plausible that they exhibit various biological targets such as other bacterial species or eukaryotic cells. Thus, biological function of *maf* genes remains a challenging question. Since these genes represent 2% of the genome of pathogenic *Neisseria*, but are virtually absent from non-pathogenic species, it is likely that they play important biological roles, including in pathogenesis. Regulation of the expression of *maf* genes could give clues on their physiological role. It has been recently demonstrated by Fagnocchi et colleagues that NadR is a regulator of *maf* operons [73]. The NadR regulon in meningococcal MC58 strain comprises *nadA* (encoding an adhesin), and the operons *NMB0375-374* (encoding MafA and MafB in MGI-1_MC58_) and *NMB0652-654* (encoding MafA, MafB and MafI in MGI-2_MC58_). In the presence of human saliva (or the small metabolite 4HPA that is secreted in human saliva) Fagnocchi et colleagues showed that *maf* genes are repressed, while *nadA* is induced in a NadR-dependent manner. This coordinate regulation indicates an adaptation of the bacteria in response to the signal molecules present in saliva, and suggests a role in colonization of the port-of-entry.

It has been suggested that, in addition to growth-inhibiting function, Rhs and Cdi might have a broader role in interbacterial communication. CdiA- or Rhs-CTs could serve as signal molecules when translocated in neighboring cells protected by the cognate immunity protein, in a manner similar to quorum sensing. Furthermore, recent studies showed that CDI plays a role in biofilm formation in *Burkholderia thailandensis* [13], [74], [75]. Indeed, CDI systems might be a mechanism allowing bacteria to discern kin versus non-kin within a complex population (*e.g.* in a polymicrobial biofilm). Only the cells expressing the same set of toxins and of immunity proteins will be able to live in close proximity. However, it is difficult to predict the evolution of a complex community since a strain may contain numerous toxin systems (such as Maf, Rhs, Cdi, bacteriocins or recently described type VI secretion systems effectors) that can all play a role in interbacterial competition. Deciphering interactions between these systems is a challenging question for future studies.

## Materials and Methods

### Bioinformatic analysis

We used 6 fully sequenced and annotated genomes of *N. meningitidis* (Z2491, MC58, FAM18 and NEM8013) and *N. gonorrhoeae* (FA1090 and NCCP11945) present in the MicroScope database with curated annotations from the NeMeSys project [19], [76]–[80]. We also used *N. gonorrhoeae* MS11 genome sequence available from the Broad Institute, *N. cinerea* ATCC 14685 genome sequenced by Washington University and the following meningococcal strains: H44/76 [81], M04-240196 [82], M01-240355 [82], G2136 [82], M6190 [82], NZ-05/33 [82], WUE2594 [83] and 053442 [20]. We used BLASTp with default parameters to search for orthologs of the Maf proteins in other Neisserial strains present in the NCBI non-redundant protein database.

To identify the different classes of MafB proteins, we downloaded 150 sequences stored on the PFAM database server [84] that have been used to build the DUF1020 (PF06255) family. The alignment was performed with ClustalW (http://www.genome.jp/tools/clustalw/) [85], [86] using default parameters and a rooted phylogenic tree (UPGMA) was generated.

Multiple alignments were also performed using MUSCLE [87] or Clustal Omega [88] with default parameters and shaded using the BoxShade server (http://www.ch.embnet.org/software/BOX_form.html). The LipoP 1.0 server [40] and SignalP server 4.1 [36] was used to predict the presence of a signal sequence with default options for Gram-negative bacteria. PRED-TAT [37], TatP 1.0 [38] or TATFIND 1.4 [39] servers were used to identify putative tat (twin arginine translocation) signal. β-barrel structure prediction were performed with TBBpred server [59] and HMM-TM server [60] and conserved domains were identified with the Conserved Domain Database (CDD) [44] of the NCBI server. Pairwise genome comparisons were visualized using Easyfig [89].

Accession numbers of proteins mentioned in the text with corresponding locus tags are listed in the supplementary information (S1 Table).

### Bacterial strains and growth conditions

All strains used in this study can be found in S2 Table.

Meningococci NEM8013 and *N. cinerea* were grown at 37°C in a moist atmosphere containing 5% CO2 on GCB (« Gonococcal Broth »; Difco) agar plates containing Kellog's supplements and appropriate antibiotics (100 µg/ml kanamycin, 6 µg/ml chloramphenicol and/or 4.5 µg/ml erythromycin for NEM8013 or 9 µg/ml erythromycin for *N. cinerea*). *E. coli* TOP10 (Life technologies) or BL21(DE3) (Life technologies) were grown at 37°C in liquid or solid Luria-Bertani (LB) medium (Difco), which contained appropriate antibiotics (50 µg/ml ticarcillin, 10 µg/ml chloramphenicol and/or 50 µg/ml kanamycin).

### Vectors construction for toxicity assays in *E. coli* BL21(DE3)

All vectors and primers used in this study can be found in S3 Table and S4 Table.

Full-length *mafB* genes including the putative signal peptide sequence or only the 5′ or 3′ regions of *mafB* genes from NEM8013 were cloned in pBAD33. Briefly, PCR products of *mafB1_MGI-2NEM8013_, mafB_MGI-1NEM8013_*, the 5′ region of *mafB_MGI-1NEM8013_* (the first 1020 nucleotides), the 3′ region of *mafB_MGI-1NEM8013_* (the last 477 nucleotides) and the 3′ region of *mafB_MGI-3NEM8013_* (the last 339 nucleotides) were digested by *Sac*I and *Xba*I, whereas PCR products of *mafB2_MGI-2NEM8013_, mafB_MGI-3NEM8013_* and the 5′ region of *mafB_MGI-3NEM8013_* (the first 1107 nucleotides) were digested by *Sma*I and *Xba*I. The digested PCR products were ligated to pBAD33 under the control of arabinose-inducible PBAD promoter. *mafB2_MGI-2NEM8013_* and *mafB1_MGI-2NEM8013_* amplified without their putative signal peptide sequence (without the first 93 nucleotides for *mafB2_MGI-2NEM8013_* and the first 120 nucleotides for *mafB1_MGI-2NEM8013_*) were also cloned in pET22 using *Bam*HI and *Xho*I to obtain proteins containing PelB peptide signal. *mafI_MGI-1NEM8013_* was cloned in pET28 using *Nco*I and *Xho*I and *mafI_MGI-3NEM8013_* was cloned in pET15 using *Xho*I and *Bam*HI. pBAD33-*mafB* or pET22-*mafB* constructs were transformed in *E. coli* BL21(DE3) with or without pET-*mafI* constructs to perform toxicity assays.

All vectors constructed were verified by PCR and sequencing.

### Vectors construction for protein production in *E. coli* BL21(DE3)

In order to product and purify MafB_MGI-1NEM8013_, the operon *mafBI_MGI-1NEM8013_* (*NMV_0410-NMV_0409*) without the sequence of the signal peptide of MafB_MGI-1NEM8013_ was cloned downstream of an IPTG inducible promoter in pET15 using *Xho*I and *Bam*HI. The resulting MafB protein harbors hexahistidine N-terminal tag.

In order to product and purify MafI_MGI-1NEM8013_, *NMV_0409* was cloned downstream of an IPTG inducible promoter in pET28 using *Nco*I and *Xho*I. The resulting MafI protein harbors hexahistidine C-terminal tag.

In order to assess the presence of MafB in the supernatant of *E. coli*, the operon *mafBI_MGI-1NEM8013_* (*NMV_0410-NMV_0409*) was cloned downstream of an IPTG inducible promoter in pET28 using *Nco*I and *Xho*I.

To assess the potential co-purification of MafB_MGI-1NEM8013_ and MafI_MGI-1NEM8013_, *mafB_MGI-1NEM8013_* and *mafI_MGI-1NEM8013_* were cloned in two different multiple cloning site (MCS) under two IPTG inducible promoters in pcolaDUET. *mafB_MGI-1NEM8013_* was cloned in MCS2 using *Bgl*II and *Kpn*I and *mafI_MGI-1NEM8013_* was cloned in MCS1 using *Bam*HI and *Hind*III.

All vectors constructed were verified by PCR and sequencing.

### Vector construction for *mafABI* deletion in *N. cinerea*

PCR reactions were used to amplify 400 bp upstream *mafA* (*NEICIv1_50108*) and 770 bp downstream *mafI* (*NEICIv1_50110*). The resulting products were digested by *Eco*RI/*Bam*HI and *Bam*HI/*Hind*III respectively and cloned into pUC19 digested by *Eco*RI/*Hind*III. A kanamycin resistance cassette *apha*-3 was then inserted in the BamHI site. The final construct containing kanamycin resistance cassette *apha-3* flanked by homologous regions for recombination was amplified by PCR using primers with DNA uptake sequence. Amplicon was introduced in *N. cinerea* by transformation and transformants were verified by PCR and sequencing.

### Vectors construction for *maf* genes expression in NEM8013 or *N. cinerea*

*mafB* genes, *mafI* genes or *mafB-mafI* operons of NEM8013 were cloned in pGCC4 under the control of an IPTG inducible promoter using *Pac*I and *Sca*I. pGCC4-*mafB* or pGCC4-*mafB-mafI* were transformed in NEM8013, pGCC4-*mafI1_MGI-2NEM8013_* was transformed in NEM8013*ctrA* [80] to insert *mafB, mafI* or *mafB-mafI* in the meningococcal intergenic region between *lctP* and *aspC*. NEM8013 strains harbouring IPTG inducible *mafB-mafI* operons have been used in competition assays. pGCC4-*mafBI_MGI-1NEM8013_* was also transformed in NEM8013*pilQ-* [90] to assess the role of PilQ on MafB secretion.

In order to express a FLAG-tagged MafB_MGI-1NEM8013_ protein in NEM8013, the 5′ region of *mafB_MGI-1NEM8013_*, with or without the sequence of its signal peptide, was amplified with a reverse primer encoding the FLAG epitope (DYKDDDDK). The resulting PCR product was cloned in pGCC4 using *Pac*I and *Sca*I. pGCC4-*mafB_MGI-1NEM8013_FLAG* or pGCC4-*mafB_MGI-1NEM8013_FLAG-SP* was transformed in NEM8013. pGCC4-*mafB_MGI-1NEM8013_FLAG* was also transformed in *N. cinerea* and in *N. cinerea ΔmafABI* to assess the role of MafA on MafB secretion.

All strains constructed were verified by PCR and sequencing.

### Toxicity assays in *E. coli*

Toxin activity was assessed by growing *E. coli* BL21(DE3) carrying pBAD33-*mafB_MGI-1NEM8013_*, pBAD33-*mafB1_MGI-2NEM8013_*, pBAD33-*mafB2_MGI-2NEM8013_* or pBAD33-*mafB_MGI-3NEM8013_* at 37°C on LB agar plates with or without 0.2% L-arabinose to induce gene expression. Growth curves were performed in LB broth at 37°C (200 rpm) and toxin expression was induced either by addition of L-arabinose to a final concentration of 0.2% (pBAD33-*mafB*) or by 1 mM IPTG (pET22- *mafB*).

To assess the protective role of *mafI*, *E. coli* BL21(DE3) were co-transformed with pBAD33-*mafB* and either cognate or non-cognate pET-*mafI*. Growth curves were performed with 0.01 mM IPTG to induce the antitoxin production throughout the experiment whereas L-arabinose (0.2%) was added 2 h post-inoculation to induce the toxin production. Growth was monitored every hour with OD 600 nm. Viability was assessed in parallel of the growth curves by spotting 5 microliters of bacterial cultures onto LB agar plates containing D-glucose (0.2%) before and after the induction of toxin expression.

### Purification of proteins

Toxin MafB_MGI-1NEM8013_ and cognate immunity protein MafI_MGI-1NEM8013_ were expressed in *E. coli* BL21 (DE3) using plasmid pET15 resulting in the production of N-terminal hexahistidine-tagged toxin and untagged immunity protein. Protein expression was induced for 2 h at 37°C with 1 mM IPTG. Proteins were purified using NiNTA metal affinity resin (Qiagen) in denaturing conditions. Bacterial pellets were lysed by sonication in lysis buffer (100 mM NaH2PO4, 10 mM TrisHCl, 8 M urea, pH 8) and centrifuged for 20 mn at 10 000 g. Supernatants were incubated with Ni-NTA resin and loaded onto columns. The resin was washed with denaturing wash buffer (100 mM NaH2PO4, 10 mM TrisHCl, 8 M urea, pH 6.3) to first remove untagged immunity protein and then, MafB_MGI-1NEM8013_ was eluted using the same buffers by lowering pH (pH 5.9 and pH 4.5). Renaturation of MafB_MGI-1NEM8013_ was achieved by serial dialysis against buffers containing decreasing concentrations of urea.

Immunity MafI_MGI-1NEM8013_ was expressed in *E. coli* BL21(DE3) using plasmid pET28 resulting in the production of a C-terminal hexahistidine-tagged immunity protein. Protein expression was induced for 1 h at 37°C with 1 mM IPTG. Proteins were purified using NiNTA resin in native conditions. Bacterial pellets were lysed by sonication in lysis buffer (50 mM NaH2PO4, 300 mM NaCl, 10 mM imidazole, pH 8) and centrifuged for 20 mn at 10 000 g. Supernatants were loaded on NiNTA resin columns. The resin was washed (50 mM NaH2PO4, 300 mM NaCl, 20 mM and 50 mM imidazole, pH 8) and MafI_MGI-1NEM8013_ was eluted using a buffer containing 250 mM imidazole (pH 8).

MafB_MGI-1NEM8013_ and MafI_MGI-1NEM8013_ were co-expressed in *E. coli* BL21(DE3) using pcolaDUET. Protein expression was induced for 2 h at 37°C with 1 mM IPTG. Proteins were purified using NiNTA resin in native conditions as described above for MafI_MGI-1NEM8013_ alone. Bound complexes were eluted with native elution buffer containing 250 mM imidazole (pH 8) for SDS-PAGE analysis and immunoblotting.

### In vivo RNase activity

*E. coli* Top10 carrying pBAD33 or pBAD33-*mafB_MGI-1NEM8013_* were grown in LB containing chloramphenicol at 37°C, 200 rpm, until OD_600_ reached 0.2, then L-Arabinose was added to a final concentration of 0.2%. Total RNA from *E. coli* was isolated before induction (T0) and 30 min after addition of L-arabinose using TRIzol reagent (Life Technologies) according to manufacturer's instructions. Total RNAs were analyzed by denaturing gel electrophoresis (5% polyacrylamide/8 M urea) and visualized by staining with ethidium bromide.

### In vitro RNase activity

Purified MafB_MGI-1NEM8013_-His_6_ alone (3 µM) and/or MafI_MGI-1NEM8013_-His_6_ (100 µM) were incubated with 4 µg of total RNA isolated from different sources with TRIzol reagent method (*N. meningitidis* NEM8013, *E. coli* TOP10 and human epithelial cells FaDu). Each reaction was performed for 30 min at 37°C in Tris-EDTA buffer and run on native 1% agarose gels containing ethidium bromide. To assess the role of divalent cations, buffers containing Mg^2+^ (10 mM Tris-HCl, 2.5 mM MgCl2) or Mn^2+^ (25 mM HEPES pH 7.4, 50 mM NaCl, 5 mM MnCl2, 1 mM DTT) have been used instead of Tris-EDTA buffer.

To assess the ability of MafB_MGI-1NEM8013_ to cleave synthetic RNA *in vitro*, purified MafB_MGI-1NEM8013_-His_6_ alone (3 µM) and/or MafI_MGI-1NEM8013_-His_6_ (100 µM) were incubated with 3 µM of a synthetic oligoribonucleotide in Tris-EDTA buffer for 15 min at 37°C. Two synthetic oligoribonucleotides (synthetized by Integrated DNA Technologies) were used: 5′-CCUGGUUUUUAAGGAGUGUCGCCAGAGUGCCGCGAAUGAAAAA -3′ (mRNA U) and 5′- CCAGGAAAAAAAGGAGAGACGCCAGAGAGCCGCGAAAGAAAAA-3′ (mRNA 0). The reactions were stopped by the addition of an equal volume of Gel Loading Buffer II (95% formamide, 18 mM EDTA, 0.025% SDS; Ambion) and incubation for 5 min at 95°C. The reaction products were separated by electrophoresis in 14% polyacrylamide/8 M urea and were visualized by ethidium bromide staining.

### Immunoblotting

Preparation of protein samples, SDS-PAGE separation, transfer to membranes and immunoblotting were performed using standard molecular biology techniques. Proteins were quantified using NanoDrop, following manufacturer's instructions.

We raised polyclonal antibodies in rabbits against purified recombinant protein MafI_MGI-1NEM8013_ and against a synthetic peptide (KNSNIHEKNYGRD) of the COOH-terminal region of MafB_MGI-1NEM8013_ protein (Proteogenix). We used rabbit polyclonal anti-FLAG antibody directed against DYKDDDDK epitope (Cell Signaling Technology) and mouse monoclonal antibody directed against NADP-dependent glutamate dehydrogenase. Bound primary antibodies were detected by goat Anti-Rabbit or Anti-Mouse HRP-linked antibodies (Cell Signaling Technology) using ECL Plus detection reagents (Pierce).

### Preparation of supernatants from meningococcal and *E. coli* cultures

Overnight cultures of *N. meningitidis* grown on GCB agar plates were used to inoculate RPMI 1640 medium (PAA) containing Kellog's supplements and 1 mM IPTG. Overnight cultures of BL21(DE3) grown on LB agar plates were used to inoculate LB medium containing appropriate antibiotic. When OD 600 reached 0.2, IPTG was added to a final concentration of 1 mM. When OD 600 reached 0.5, the cells were harvested by centrifugation (3 000× *g* for 30 min), and supernatants were passed through a 0.22-µm pore size filter unit. Supernatant proteins were concentrated by ultrafiltration (Amicon, Ultra-15, 3 kDa cutoff) according to the manufacturer's instructions. The pellets and the concentrated supernatants were used for immunoblotting analysis.

### Competition assays

Overnight cultures of putative target cells and putative inhibitory cells grown on GCB agar plates were used to inoculate Ham's F12 (PAA) containing Kellog's supplements and 1 mM IPTG. Putative target cells were an unencapsulated derivative of NEM8013 with a transposon insertion in the *ctrA* gene [80] or this unencapsulated derivative overexpressing MafI1_MGI-2NEM8013_. Putative inhibitory cells were NEM8013 overexpressing each of the four *mafB* toxins and their cognate *mafI* immunity genes. When OD 600 reached 0.8, cultures were mixed at an inhibitor to target cell ratio of 10 to 1 (∼8×10^9^ inhibitor cells and ∼8×10^8^ target cells) and centrifuged at 4 000 rpm for 5 min. 10 µl of the mixed culture pellet were spotted on a membrane filter (pore size, 0.45 µm) placed on GCB agar plate containing 1 mM IPTG and incubated overnight. 10 µl of mixed cultures were also used for determination of the inhibitor/target initial ratio by plating on GCB with appropriate antibiotics. Filters recovered after overnight incubation were used to perform viable counts and the competitive index (CI) was calculated as the inhibitor/target ratio in the output divided by the initial inhibitor/target ratio. The data from three independent experiments were examined for significance using a two-tailed Student's t-test. A *p*-value *p*<*0.05* was considered significant.

## Supporting Information

S1 Fig **Amino acid alignment of class 1 MafBs.** Protein sequences were aligned using Clustal Omega (1.2.1), with default parameters and shaded using the BoxShade server. Residues that are identical or similar in all sequences are shaded with black or grey background respectively. The GenBank locus tag was used to identify each protein. The end of the conserved N terminal region is aligned to show the VSGDF motif which demarcates the beginning of the variable C terminal region in NMB0653, NMV_1757, NMA2113, NMB0374 or the beginning of the bacterial intein in NGO1392, NGO1585, NGK_1637, NGK_1886.
(TIF)

S2 Fig **Amino acid alignment of class 2 MafBs.** Protein sequences were aligned using Clustal Omega (1.2.1), with default parameters, and shaded using the BoxShade server. Residues that are identical or similar in all sequences are shaded with black or grey background respectively. The GenBank locus tag was used to identify each protein. The end of the conserved N terminal region is aligned to show the VKYDT motif that demarcates the beginning of the variable C terminal region.
(TIF)

S3 Fig **Amino acid alignment of class 3 MafBs.** Protein sequences were aligned using Clustal Omega (1.2.1), with default parameters and shaded using the BoxShade server. Residues that are identical or similar in all sequences are shaded with black or grey background respectively. The GenBank locus tag was used to identify each protein. The end of the conserved N terminal region is aligned to show the WDWVKN motif that demarcates the beginning of the variable C terminal region.
(TIF)

S4 Fig **MafB1_MGI-2NEM8013_ and MafB2_MGI-2NEM8013_ are not toxic in** ***E. coli*** **periplasm and the toxicity of MafB_MGI-1NEM8013_ resides in its C-terminal domain.** A) Growth curves of BL21(DE3) cells transformed with vector pET22 carrying *mafB1_MGI-2NEM8013_* or *mafB2_MGI-2NEM8013_* genes. Toxin expression was induced by adding 1 mM IPTG in LB broth 2 h after inoculation (arrow). B) Effect of MafB_MGI-1NEM8013_ N-terminal domain over-expression on *E. coli* grown in the presence of 0.2% L-arabinose (Ara+). BL21(DE3) cells were transformed with vector pBAD33 carrying the 5′end of *mafB_MGI-1NEM8013_* gene. C) Effect of MafB_MGI-1NEM8013_ C-terminal domain over-expression on *E. coli* grown in the presence of 0.2% L-arabinose (Ara+). BL21(DE3) cells were transformed with vector pBAD33 carrying the 3′end of *mafB_MGI-1NEM8013_* gene. Inhibition of growth due to the toxin is counteracted by cognate immunity protein co-expression. LB agar plates contain 0.01 mM IPTG to induce expression of *mafI_MGI-1NEM8013_* cloned in pET28. Control strains contain empty vectors.
(TIF)

S5 Fig **MafB_MGI-1NEM8013_ is a secreted toxin.** MafB_MGI-1NEM8013_ (MafB) is detected in the whole-cell lysates (WC) and in the supernatant of NEM8013 strain expressing MafB_MGI-1NEM8013_ and MafI_MGI-1NEM8013_ under an IPTG inducible promoter. NEM8013 parental strain is used as a control (ctrl). When the background strain was a *pilQ-* derivative of NEM8013, MafB_MGI-1NEM8013_ was also detected in the supernatant. Antibodies used for immunoblotting of WC and supernatants were Anti-NADP-GDH (NADP-dependent glutamate dehydrogenase, as a cytoplasmic marker protein) and an Anti-peptide that recognizes a C-terminal epitope of MafB_MGI-1NEM8013_.
(TIF)

S6 Fig **MafA is not required for MafB secretion and MafB is not secreted by** ***E. coli*** **.** A) C-terminal FLAG-tagged MafB_MGI-1NEM8013_ (MafB) is detected in the whole-cell lysate (WC) and in the supernatant of *N. cinerea* strain (Nc) expressing MafB under an IPTG inducible promoter. This IPTG inducible construction has been inserted in an intergenic region of *N. cinerea* chromosome using pGCC4 vector. *N. cinerea* parental strain is used as a control. B) MafB_MGI-1NEM8013_ is detected in the whole-cell lysate and in the supernatant of a *N. cinerea* mutant where the whole *mafABI* locus (MGI-1_Nc14685_) has been replaced by a kanamycin resistance cassette. Antibodies used for immunoblotting of whole-cell lysates and supernatants were Anti-NADP-GDH (NADP-dependent glutamate dehydrogenase, as a cytoplasmic marker protein) and Anti-FLAG to detect C-terminal, FLAG-tagged MafB. A non-specific band detected with Anti-NADP-GDH antibody is indicated with an arrow. C) MafB_MGI-1NEM8013_ is detected in the whole-cell lysate but not in the supernatant of *E. coli* BL21(DE3) transformed with pET28*mafBI*_MGI-1NEM8013_. Antibodies used for immunoblotting of whole-cell lysates and supernatants were Anti-MBP (Maltose Binding Protein, as a periplasmic marker protein) and an Anti-peptide that recognizes a N-terminal epitope of MafB_MGI-1NEM8013_.
(TIF)

S1 Table **Correspondence between the new nomenclature and the old one for** ***maf*** **genes in the 6 genomes used for pairwise comparison.**
(DOC)

S2 Table **Strains used in this study.**
(DOC)

S3 Table **Vectors used in this study.**
(DOC)

S4 Table **Oligonucleotides used in this study.**
(DOC)
