# Supplementary figures and images for: A New Family of Secreted Toxins in Pathogenic Neisseria Species
Source: PLoS Pathog. 2015 Jan 8;11(1):e1004592. doi: 10.1371/journal.ppat.1004592 (PMC4287609; doi:10.1371/journal.ppat.1004592)

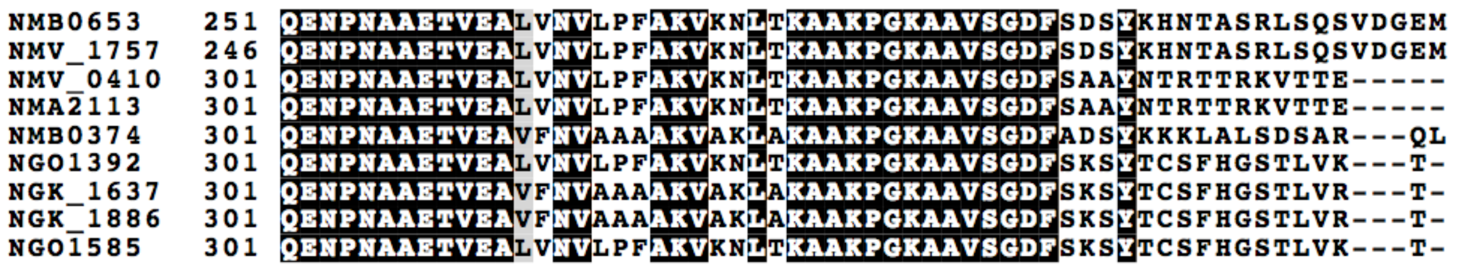

Supplement: S1 Fig — Amino acid alignment of class 1 MafBs. Protein sequences were aligned using Clustal Omega (1.2.1), with default parameters and shaded using the BoxShade server. Residues that are identical or similar in all sequences are shaded with black or grey background respectively. The GenBank locus tag was used to identify each protein. The end of the conserved N terminal region is aligned to show the VSGDF motif which demarcates the beginning of the variable C terminal region in NMB0653, NMV_1757, NMA2113, NMB0374 or the beginning of the bacterial intein in NGO1392, NGO1585, NGK_1637, NGK_1886. (TIF) [file ppat.1004592.s001.tif]

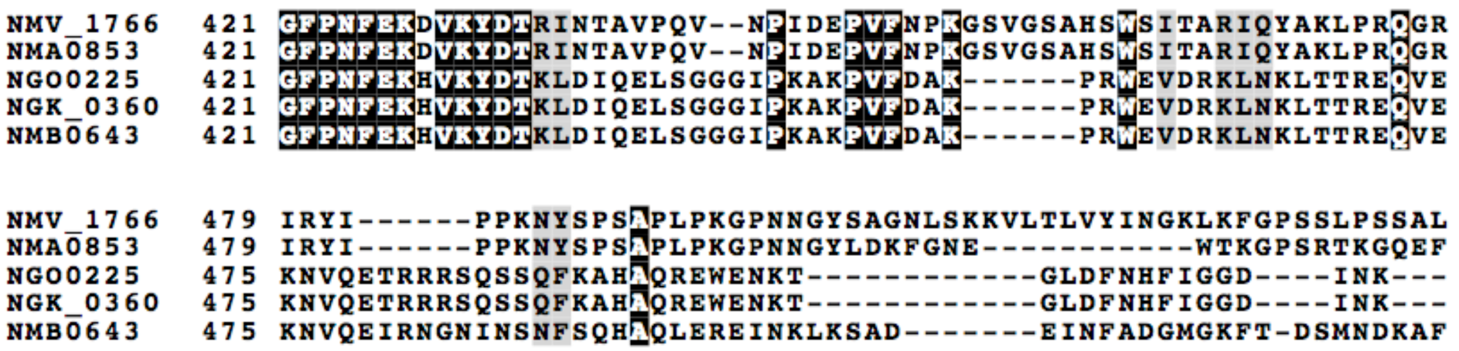

Supplement: S2 Fig — Amino acid alignment of class 2 MafBs. Protein sequences were aligned using Clustal Omega (1.2.1), with default parameters, and shaded using the BoxShade server. Residues that are identical or similar in all sequences are shaded with black or grey background respectively. The GenBank locus tag was used to identify each protein. The end of the conserved N terminal region is aligned to show the VKYDT motif that demarcates the beginning of the variable C terminal region. (TIF) [file ppat.1004592.s002.tif]

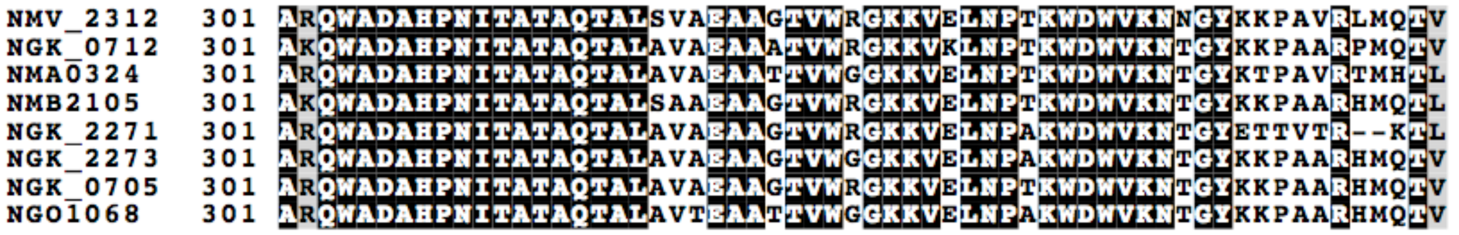

Supplement: S3 Fig — Amino acid alignment of class 3 MafBs. Protein sequences were aligned using Clustal Omega (1.2.1), with default parameters and shaded using the BoxShade server. Residues that are identical or similar in all sequences are shaded with black or grey background respectively. The GenBank locus tag was used to identify each protein. The end of the conserved N terminal region is aligned to show the WDWVKN motif that demarcates the beginning of the variable C terminal region. (TIF) [file ppat.1004592.s003.tif]

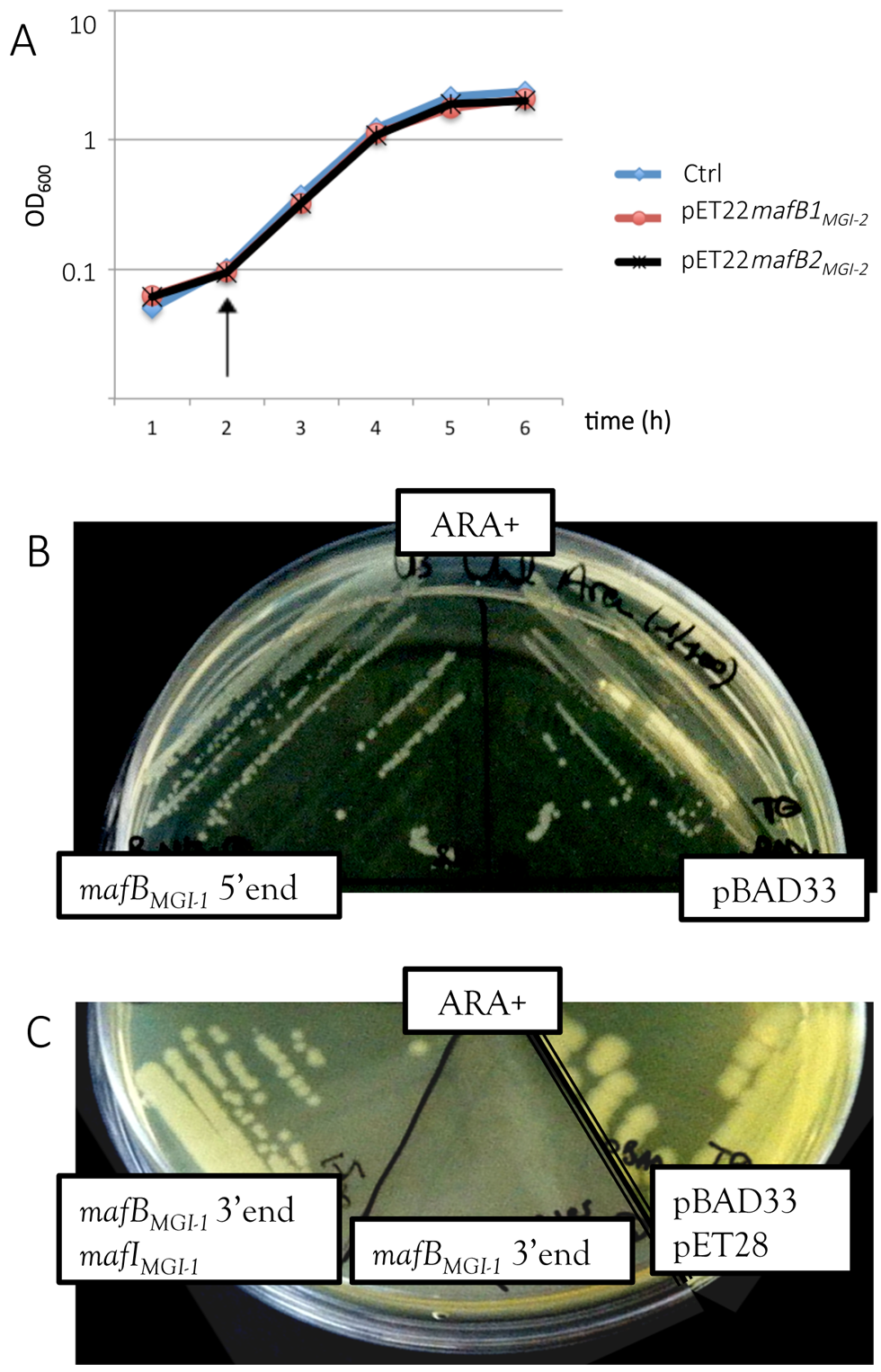

Supplement: S4 Fig — MafB1MGI-2NEM8013 and MafB2MGI-2NEM8013 are not toxic in E. coli periplasm and the toxicity of MafBMGI-1NEM8013 resides in its C-terminal domain. A) Growth curves of BL21(DE3) cells transformed with vector pET22 carrying mafB1MGI-2NEM8013 or mafB2MGI-2NEM8013 genes. Toxin expression was induced by adding 1 mM IPTG in LB broth 2 h after inoculation (arrow). B) Effect of MafBMGI-1NEM8013 N-terminal domain over-expression on E. coli grown in the presence of 0.2% L-arabinose (Ara+). BL21(DE3) cells were transformed with vector pBAD33 carrying the 5′end of mafBMGI-1NEM8013 gene. C) Effect of MafBMGI-1NEM8013 C-terminal domain over-expression on E. coli grown in the presence of 0.2% L-arabinose (Ara+). BL21(DE3) cells were transformed with vector pBAD33 carrying the 3′end of mafBMGI-1NEM8013 gene. Inhibition of growth due to the toxin is counteracted by cognate immunity protein co-expression. LB agar plates contain 0.01 mM IPTG to induce expression of mafIMGI-1NEM8013 cloned in pET28. Control strains contain empty vectors. (TIF) [file ppat.1004592.s004.tif]

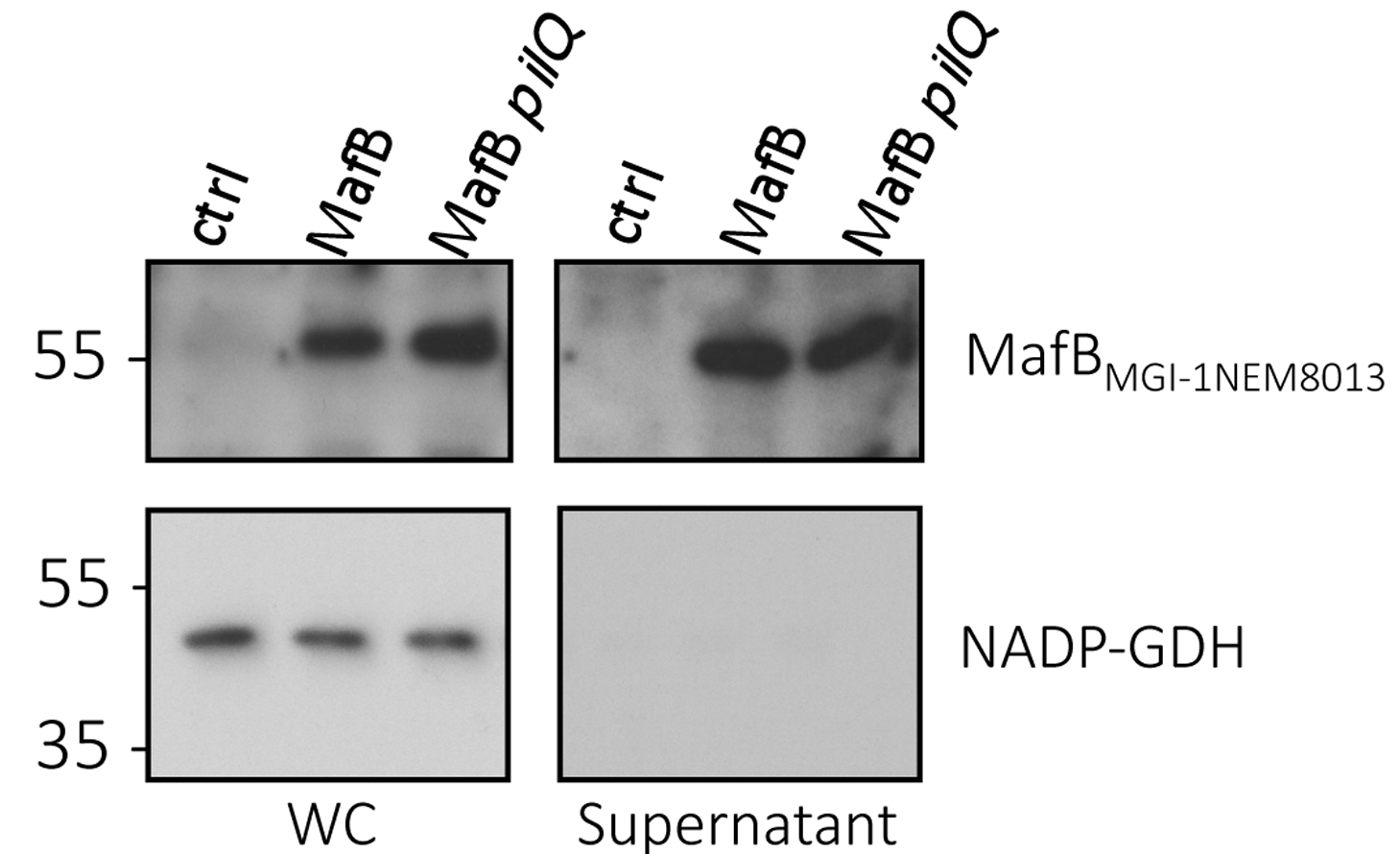

Supplement: S5 Fig — MafBMGI-1NEM8013 is a secreted toxin. MafBMGI-1NEM8013 (MafB) is detected in the whole-cell lysates (WC) and in the supernatant of NEM8013 strain expressing MafBMGI-1NEM8013 and MafIMGI-1NEM8013 under an IPTG inducible promoter. NEM8013 parental strain is used as a control (ctrl). When the background strain was a pilQ- derivative of NEM8013, MafBMGI-1NEM8013 was also detected in the supernatant. Antibodies used for immunoblotting of WC and supernatants were Anti-NADP-GDH (NADP-dependent glutamate dehydrogenase, as a cytoplasmic marker protein) and an Anti-peptide that recognizes a C-terminal epitope of MafBMGI-1NEM8013. (TIF) [file ppat.1004592.s005.tif]

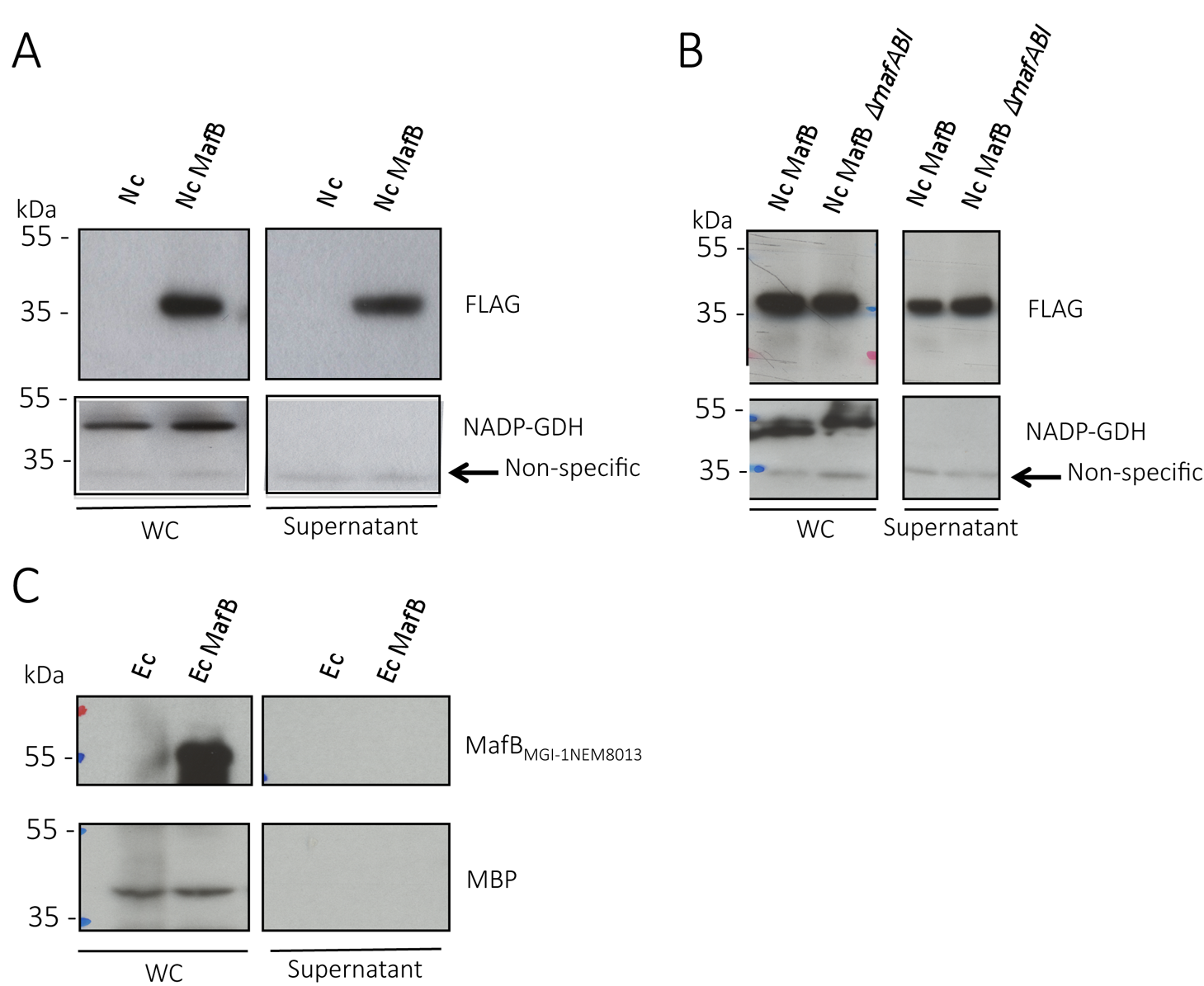

Supplement: S6 Fig — MafA is not required for MafB secretion and MafB is not secreted by E. coli . A) C-terminal FLAG-tagged MafBMGI-1NEM8013 (MafB) is detected in the whole-cell lysate (WC) and in the supernatant of N. cinerea strain (Nc) expressing MafB under an IPTG inducible promoter. This IPTG inducible construction has been inserted in an intergenic region of N. cinerea chromosome using pGCC4 vector. N. cinerea parental strain is used as a control. B) MafBMGI-1NEM8013 is detected in the whole-cell lysate and in the supernatant of a N. cinerea mutant where the whole mafABI locus (MGI-1Nc14685) has been replaced by a kanamycin resistance cassette. Antibodies used for immunoblotting of whole-cell lysates and supernatants were Anti-NADP-GDH (NADP-dependent glutamate dehydrogenase, as a cytoplasmic marker protein) and Anti-FLAG to detect C-terminal, FLAG-tagged MafB. A non-specific band detected with Anti-NADP-GDH antibody is indicated with an arrow. C) MafBMGI-1NEM8013 is detected in the whole-cell lysate but not in the supernatant of E. coli BL21(DE3) transformed with pET28mafBI MGI-1NEM8013. Antibodies used for immunoblotting of whole-cell lysates and supernatants were Anti-MBP (Maltose Binding Protein, as a periplasmic marker protein) and an Anti-peptide that recognizes a N-terminal epitope of MafBMGI-1NEM8013. (TIF) [file ppat.1004592.s006.tif]
