# Supplementary material for: A New Family of Secreted Toxins in Pathogenic Neisseria Species
Source: PLoS Pathog. 2015 Jan 8;11(1):e1004592. doi: 10.1371/journal.ppat.1004592 (PMC4287609; doi:10.1371/journal.ppat.1004592)
Supplement: S1 Table — Correspondence between the new nomenclature and the old one for maf genes in the 6 genomes used for pairwise comparison. (DOC) [file ppat.1004592.s007.doc]

**Table S1. Correspondence between the new nomenclature and the old one for *maf* genes in the 6 genomes used for pairwise comparison.**

| Locus tag | NeMeSys annotation | Predicted function | Locus | Proposed nomenclature | Accession number | Class |
| --- | --- | --- | --- | --- | --- | --- |
| **NEM8013** |  |  |  |  | FM999788 |  |
| NMV_0410 | mafB1 | Toxin | MGI-1 | mafB_MGI-1NEM8013_ | CAX49352 | 1 |
| NMV_0409 | - | Immunity | MGI-1 | mafI_MGI-1NEM8013_ | CAX49351 |  |
| NMV_1757 | mafB2 | Toxin | MGI-2 | mafB2_MGI-2NEM8013_ | CAX50568 | 1 |
| NMV_1756 | - | Immunity | MGI-2 | mafI2_MGI-2NEM8013_ | CAX50567 |  |
| NMV_1766 | mafB-related | Toxin | MGI-2 | mafB1_MGI-2NEM8013_ | CAX50576 | 2 |
| NMV_1765 | - | Immunity | MGI-2 | mafI1_MGI-2NEM8013_ | CAX50575 |  |
| NMV_2312 | mafB3 | Toxin | MGI-3 | mafB_MGI-3NEM8013_ | CAX51064 | 3 |
| NMV_2313 | - | Immunity | MGI-3 | mafI_MGI-3NEM8013_ | CAX51065 |  |
| **MC58** |  |  |  |  | AE002098 |  |
| NMB0374 | mafB1 | Toxin | MGI-1 | mafB_MGI-1MC58_ | AAF62308 | 1 |
| NMB0373 | - | Immunity | MGI-1 | mafI_MGI-1MC58_ | AAF40815 |  |
| NMB0653 | mafB2 | Toxin | MGI-2 | mafB2_MGI-2MC58_ | AAF62314 | 1 |
| NMB0654 | - | Immunity | MGI-2 | mafI2_MGI-2MC58_ | AAF41074 |  |
| NMB0643 | mafB-related | Toxin | MGI-2 | mafB1_MGI-2MC58_ | AAF62313 | 2 |
| NMB0644 | - | Immunity | MGI-2 | mafI1_MGI-2MC58_ | AAF41066 |  |
| NMB2105 | mafB3 | Toxin | MGI-3 | mafB_MGI-3MC58_ | AAF62340 | 3 |
| NMB2106 | - | Immunity | MGI-3 | mafI_MGI-3MC58_ | AAF42421 |  |
| **Z2491** |  |  |  |  | AL157959 |  |
| NMA2113 | mafB2 | Toxin | MGI-1 | mafB_MGI-1Z2491_ | CAM09212 | 1 |
| NMA2114 | - | Immunity | MGI-1 | mafI_MGI-1Z2491_ | CAM09213 |  |
| NMA0853 | mafB-related | Toxin | MGI-2 | mafB_MGI-2Z2491_ | CAM08090 | 2 |
| NMA0854 | - | Immunity | MGI-2 | mafI_MGI-2Z2491_ | CAM08091 |  |
| NMA0324 | mafB1 | Toxin | MGI-3 | mafB_MGI-3Z2491_ | CAM07626 | 3 |
| NMA0323 | - | Immunity | MGI-3 | mafI_MGI-3Z2491_ | CAM07625 |  |
| **FA1090** |  |  |  |  | AE004969 |  |
| NGO1585 | mafB3 | Toxin | MGI-1 | mafB_MGI-1FA1090_ | AAW90214 | 1 |
| NGO1586 | - | Immunity | MGI-1 | mafI_MGI-1FA1090_ | AAW90215 |  |
| NGO0225 | mafB-related | Toxin | MGI-2 | mafB_MGI-2FA1090_ | AAW88978 | 2 |
| NGO0226 | - | Immunity | MGI-2 | mafI_MGI-2FA1090_ | AAW88979 |  |
| NGO1971 | mafB4 | Toxin | MGI-3 | mafB_MGI-3FA1090_ | AAW90580 | 3 |
| NGO1970 | - | Immunity | MGI-3 | mafI_MGI-3FA1090_ | AAW90579 |  |
| NGO1068 | mafB1 | Toxin | MGI-4 | mafB_MGI-4FA1090_ | AAW89738 | 3 |
| NGO1069 | - | Immunity | MGI-4 | mafI_MGI-4FA1090_ | AAW89739 |  |
| NGO1392 | mafB2 | Toxin | MGI-5 | mafB_MGI-5FA1090_ | AAW90040 | 1 |
| NGO1391 | - | Immunity | MGI-5 | mafI_MGI-5FA1090_ | AAW90039 |  |
| **NCCP11945** |  |  |  |  | CP001050 |  |
| NGK_1886 | mafB4 | Toxin | MGI-1 | mafB_MGI-1NCCP11945_ | ACF30524 | 1 |
| NGK_1887 | - | Immunity | MGI-1 | mafI_MGI-1NCCP11945_ | ACF30525 |  |
| NGK_0360 | mafB-related | Toxin | MGI-2 | mafB_MGI-2NCCP11945_ | ACF29053 | 2 |
| NEIGN0292 | - | Immunity | MGI-2 | mafI_MGI-2NCCP11945_ | - |  |
| NGK_2271 | mafB5 | Toxin | MGI-3 | mafB1_MGI-3NCCP11945_ | ACF30875 | 3 |
| NGK_2272 | - | Immunity | MGI-3 | mafI1_MGI-3NCCP11945_ | ACF30876 |  |
| NGK_2273 | mafB6 | Toxin | MGI-3 | mafB2_MGI-3NCCP11945_ | ACF30877 | 3 |
| NGK_2274 | - | Immunity | MGI-3 | mafI2_MGI-3NCCP11945_ | ACF30878 |  |
| NGK_0712 | mafB2 | Toxin | MGI-4 | mafB1_MGI-4NCCP11945_ | ACF29394 | 3 |
| NGK_0711 | - | Immunity | MGI-4 | mafI1_MGI-4NCCP11945_ | ACF29393 |  |
| NGK_0705 | mafB1 | Toxin | MGI-4 | mafB2_MGI-4NCCP11945_ | ACF29387 | 3 |
| NEIGN0607 | - | Immunity | MGI-4 | mafI2_MGI-4NCCP11945_ | - |  |
| NGK_1637 | mafB3 | Toxin | MGI-5 | mafB_MGI-5NCCP11945_ | ACF30287 | 1 |
| NGK_1636 | - | Immunity | MGI-5 | mafI_MGI-5NCCP11945_ | ACF30286 |  |
| **ATCC 14685** |  |  |  |  | ACDY00000000 |  |
| NEICIv1_50109 | - | Toxin | MGI-1 | mafB_MGI-1Nc14685_ | WP_003676498 | 1 |
| NEICIv1_50110 | - | Immunity | MGI-1 | mafI_MGI-1Nc14685_ | WP_003676501 |  |
