# Supplementary material for: A New Family of Secreted Toxins in Pathogenic Neisseria Species
Source: PLoS Pathog. 2015 Jan 8;11(1):e1004592. doi: 10.1371/journal.ppat.1004592 (PMC4287609; doi:10.1371/journal.ppat.1004592)
Supplement: S2 Table — Strains used in this study. (DOC) [file ppat.1004592.s008.doc]

**Table S2. Strains used in this study**

| **Strains** | **Description** | **Reference** |
| --- | --- | --- |
| NEM8013 | Sequenced strain *N. meningitidis* serogroup C | Rusniok, 2009 |
| NEM8013*ctrA* | Unencapsulated derivative of NEM8013. Insertion of a transposon in *ctrA* (Kanamycin resistance) | Rusniok, 2009 |
| NEM8013*pilQ* | Insertion of a transposon in *pilQ* (Kanamycin resistance) | Hélaine, 2005 |
| *N. cinerea* ATCC 14685 | Sequenced strain of *N. cinerea* | Weinstock G (Washington University Genome Sequencing Center) |
| *E. coli* TOP10 | - | Life technologies |
| *E. coli* BL21(DE3) | - | Life technologies |
