# Supplementary material for: A New Family of Secreted Toxins in Pathogenic Neisseria Species
Source: PLoS Pathog. 2015 Jan 8;11(1):e1004592. doi: 10.1371/journal.ppat.1004592 (PMC4287609; doi:10.1371/journal.ppat.1004592)
Supplement: S3 Table — Vectors used in this study. (DOC) [file ppat.1004592.s009.doc]

**Table S3. Vectors used in this study**

| **Vectors** | **Description** | **Reference** |
| --- | --- | --- |
| pBAD33 | Arabinose inducible promoter | Guzman, 1995 |
| pET15 | IPTG inducible promoter, N-terminal His6 tag | Novagen |
| pET28 | IPTG inducible promoter, C-terminal His6 tag | Novagen |
| pET22 | IPTG inducible promoter, N-terminal PelB signal peptide | Novagen |
| pcolaDUET | IPTG inducible promoter, 2 cloning sites, N-terminal His6 tag (MCS1) and C-terminal S-tag (MCS2) | Novagen |
| pGCC4 | Insertion of a sequence between lctP and aspC genes of Neisseria sp., IPTG inducible promoter | Mehr, 2000 |
