# Supplementary material for: A New Family of Secreted Toxins in Pathogenic Neisseria Species
Source: PLoS Pathog. 2015 Jan 8;11(1):e1004592. doi: 10.1371/journal.ppat.1004592 (PMC4287609; doi:10.1371/journal.ppat.1004592)
Supplement: S4 Table — Oligonucleotides used in this study. (DOC) [file ppat.1004592.s010.doc]

**Table S4. Oligonucleotides used in this study**

| **Oligonucleotides** | **Sequences (5'-3')** | **Vector** | **Comments** |
| --- | --- | --- | --- |
| ***mafB*** |  |  |  |
| SacINMV1766pBAD33 | CCCCGAGCTCAGGAGGGAGTATTGGGCATTTCCCGCAAAATATCC | pBAD33 | used to amplify NMV_1766 for cloning into pBAD33 |
| XbaINMV1766pBAD33 | CCCCTCTAGATTATCTAGCAGGGAATTTAAATTTTAAC | pBAD33 | used to amplify NMV_1766 for cloning into pBAD33 |
| SacINMV410pBAD33 | CCCCGAGCTCAGGAGGGAGTAGTGAAACCGCTGCGAAGACTC | pBAD33 | used to amplify NMV_0410 for cloning into pBAD33 |
| XbaINMV410pBAD33 | CCCCtctagaTTATTTTACAGGGTTATATTTGG | pBAD33 | used to amplify NMV_0410 for cloning into pBAD33 |
| FSacI_mafB1Cterm | CCCCGAGCTCAGGAGGGAGTAATGTCTGCTGCATACAATAC | pBAD33 | used to amplify 3' extremity of NMV_0410 for cloning into pBAD33 |
| RXbaI_mafB1Nter | CCCCTCTAGATTAaaaatccccgctaaccgcagc | pBAD33 | used to amplify 5' extremity of NMV_0410 for cloning into pBAD33 |
| NcoImafB1 | catgccatggcatgc GTGAAACCGCTGCGAAGACTCATCAAGC | pET28 | used to amplify NMV_0410 for cloning into pET28 |
| FXhoImafB1pET15bsansPS | CCGCTCGAGGACCCGTTCATTACCGATA | pET15 | used to amplify NMV_0410 (w/o Signal peptide sequence) and NMV_0409 for cloning into pET15 |
| RBamHImafI1pET15b | CCGGGATCCTTAATCTGATGTACTTTCAAT | pET15 | used to amplify NMV_0410 (w/o Signal peptide sequence) and NMV_0409 for cloning into pET15 |
| FBglIIpcolaNMV410 | CATAGATCTaGCGCAAGACCCGTTCATTACC | pcolaDUET | used to amplify NMV_0410 for cloning into pcolaDUET |
| RKpnIpcolaNMV410 | catGGTACCTTTTACAGGGTTATATTTGG | pcolaDUET | used to amplify NMV_0410 for cloning into pcolaDUET |
| RXbaINMV1757pBAD33 | CCCCTCTAGACTATTGCACCTTTTTAATGGTTTTAGGG | pBAD33 | used to amplify NMV_1757 for cloning into pBAD33 |
| FSmaINMV1757pBAD33 | CCCCCCCGGGAGGAGGGAGTAGTGAAACCGCTGCGCAGACTGACAAACCTCCTTGCCG | pBAD33 | used to amplify NMV_1757 for cloning into pBAD33 |
| RXbaINMV2312pBAD33 | CCCCTCTAGATTATTTGATGGGTAACCATGGGG | pBAD33 | used to amplify NMV_2313 for cloning into pBAD33 |
| FSmaINMV2312pBAD33 | CCCCCCCGGGAGGAGGGAGTAATGAAATTGCCTATTCAAAAATTCATGATGCTGTTTGC | pBAD33 | used to amplify NMV_2313 for cloning into pBAD33 |
| FSacI_mafB3Cterm | CCCCGAGCTCAGGAGGGAGTAATGAAGCCACCGAAACCAAG | pBAD33 | used to amplify 3' extremity of NMV_2313 for cloning into pBAD33 |
| RXbaI_mafB3Nter | CCCCTCTAGATTAATTTCCCCCAGCCATCTCC | pBAD33 | used to amplify 5' extremity of NMV_2313 for cloning into pBAD33 |
| FBamHINMV1766pET22 | CATGGGATCCCATGTCAGATTTGGCAAACGATTCTTTTATCCGGCAGGTTCTCGACCG | pET22 | used to amplify NMV_1766 (w/o Signal peptide sequence) for cloning into pET22 |
| RXhoINMV1766pET22 | CCGCTCGAGCGGTCTAGCAGGGAATTTAAATTTTAACTCTGTTGGC | pET22 | used to amplify NMV_1766 (w/o Signal peptide sequence) for cloning into pET22 |
| FBamHINMV1757pET22 | CATGGGATCCCATGCAAGACCCGTTCATTACCGATAACGCCCAACGGCAGCACTAC | pET22 | used to amplify NMV_1757 (w/o Signal peptide sequence) for cloning into pET22 |
| RXhoINMV1757pET22 | CCGCTCGAGCGGTTGCACCTTTTTAATGGTTTTAGGGTTAGCCGG | pET22 | used to amplify NMV_1757 (w/o Signal peptide sequence) for cloning into pET22 |
| FcompNMV410 | ccttaattaaggagtaattttGTGAAACCGCTGCGAAGAC | pGCC4 | used to amplify NMV_0410 for cloning into pGCC4 |
| FcompNMV410sansPS | ccttaattaaggagtaattttATGGCGCAAGACCCGTTC | pGCC4 | used to amplify NMV_0410 (w/o Signal peptide sequence) for cloning into pGCC4 |
| RcompNMV410 | cgacagtactTTATTTTACAGGGTTATATTTGG | pGCC4 | used to amplify NMV_0410 for cloning into pGCC4 |
| Rcomp410FLAG | cgacagtactTTActtgtcatcgtcatccttgtaatcAAAATCCCCGCTAACCGCAG | pGCC4 | used to amplify NMV_0410 with FLAG sequence instead of the sequence encoding C-terminal domain for cloning into pGCC4 |
| RcompNMV409 | cgacagtactttattaatctgatgtactttcaattcc | pGCC4 | used to amplify NMV_0409 and NMV_0410 for cloning into pGCC4 |

| FcompmafBrel | ccttaattaaggagtaattttTTGGGCATTTCCCGCAAAATATCC | pGCC4 | used to amplify NMV_1766 for cloning into pGCC4 |
| --- | --- | --- | --- |
| RcompmafBrel | cgacagtactTTATCTAGCAGGGAATTTAAATTTTAAC | pGCC4 | used to amplify NMV_1766 for cloning into pGCC4 |
| RcompNMV1765 | cgacagtacttcaggtgccgaggtttaaag | pGCC4 | used to amplify NMV_1765 and NMV_1766 for cloning into pGCC4 |
| FcompNMV2312B3 | ccttaattaaggagtaattttatgATGAAATTGCCTATTC | pGCC4 | used to amplify NMV_2312 for cloning into pGCC4 |
| RcompNMV2312B3 | cgacagtactttaTTTGATGGGTAACC | pGCC4 | used to amplify NMV_2312 for cloning into pGCC4 |
| Rcomp2312FLAGscaI | cgacagtactTTActtgtcatcgtcatccttgtaatcATTTCCCCCAGCCATCTCC | pGCC4 | used to amplify NMV_2312 with FLAG sequence instead of the sequence encoding C-terminal domain for cloning into pGCC4 |
| RcompNMV2313 | cgacagtactttaTTTGAGTATATTG | pGCC4 | used to amplify NMV_2313 and NMV_2312 for cloning into pGCC4 |
| Fcomp1757_mafB2PacI | CCTTAATTAAGGAGTAATTTTGTGAAACCGCTGCGCAGACTG | pGCC4 | used to amplify NMV_1757 for cloning into pGCC4 |
| Rcomp1757_mafB2PacI | CCTTAATTAACTATTGCACCTTTTTAATGG | pGCC4 | used to amplify NMV_1757 for cloning into pGCC4 |
| Rcomp1756_mafI2PacI | CCTTAATTAACTACGCTTGCGAAATTAATTCC | pGCC4 | used to amplify NMV_1756 and NMV_1757 for cloning into pGCC4 |
| ***mafI*** |  |  |  |
| FcompNMV1765 | ccttaattaaggagtaattttatgAATTTAGAGACAGCAG | pGCC4 | used to amplify NMV_1765 for cloning into pGCC4 |
| RcompNMV1765 | cgacagtacttcaggtgccgaggtttaaag | pGCC4 | used to amplify NMV_1765 for cloning into pGCC4 |
| FXhoINMV2313pET15b | CCGCTCGAGATGATGACGGAACCCCATGGTTACCC | pET15 | used to amplify NMV_2313 for cloning into pET15 |
| RBamHINMV2313pET15b | CCGGGATCCTTATTTGAGTATATTGTCAAATTC | pET15 | used to amplify NMV_2313 for cloning into pET15 |
| NcoInmv409 | catgccatggcatgcATGAAAACATTAGACGAACG | pET28 | used to amplify NMV_0409 for cloning into pET28 |
| XhoInmv409 | ccgctcgagcgggtaatctgatgtactttcaattcc | pET28 | used to amplify NMV_0409 for cloning into pET28 |
| FbamhIpcolaNMV409 | CATGGGATCCGATGAAAACATTAGACGAACG | pcolaDUET | used to amplify NMV_0409 for cloning into pcolaDUET |
| RhindIIIpcolaNMV409 | atcaagcttttaatctgatgtactttcaattcc | pcolaDUET | used to amplify NMV_0409 for cloning into pcolaDUET |
| **delta *NMV_1756*** |  |  |  |
| FupNMV1756EcoRI | AtcgaattCAGACTCCTACAAGCATAAC | pUC19 | used to amplify region upstream NMV_1756 for cloning into pUC19 |
| RupNMV1756BamHI | AtcggatccATTCATAACTATTGCACC | pUC19 | used to amplify region upstream NMV_1756 for cloning into pUC19 |
| FdownNMV1756BamHI | AtcggatccAATGGTGCAGTATTCAGGAG | pUC19 | used to amplify region downstream NMV_1756 for cloning into pUC19 |
| RdownNMV1756HindIII | AtcaagcttGGAAGGAGCAGGTGTAGG | pUC19 | used to amplify region downstream NMV_1756 for cloning into pUC19 |
| **delta *mafABI N. cinerea*** |  |  |  |
| FupNcinmafAEcoRI | AtcgaattcCGTTTACGACGCCGTCTCAC | pUC19 | used to amplify region upstream mafA for cloning into pUC19 |
| RupNcinmafABamHI | AtcggatccAGCGGAGTTTGTTTGATGTTG | pUC19 | used to amplify region upstream mafA for cloning into pUC19 |
| FdownNcinmafIBamHI | AtcggatccCATAGTCAACGTGCAATTTG | pUC19 | used to amplify region downstream mafI for cloning into pUC19 |
| RdownNcinmafIHindIII | AtcaagcttTTAACGACCGCGTCGACCATG | pUC19 | used to amplify region downstream mafI for cloning into pUC19 |
| Fk7kanaTopoBamHI | GCCGGATCCAATGAGCTGATTTAAC | pUC19 | used to amplify apha3 for cloning into pUC19 |
| Rk7kanaTopoBamHI | GCCGGATCCTCAGAAGAACTCGTCAAGAAGG | pUC19 | used to amplify apha3 for cloning into pUC19 |
| DUSFupNcinmafA | ATCGCCGTCTGAACGTTTACGACGCCGTCTCAC | - |  |
| DUSRdNcinmafI | AtcTTCAGACGGCTTAACGACCGCGTCGACCATG | - |  |
